# Supplementary material for: Exploring parents’ views of the use of narratives to promote childhood vaccination online
Source: PLoS One. 2023 Jul 19;18(7):e0284107. doi: 10.1371/journal.pone.0284107 (PMC10355395; doi:10.1371/journal.pone.0284107)
Supplement: S1 File — (DOCX) [file pone.0284107.s001.docx]

**Information about the videos**

Videos used for this project were not created for our specific project but were identified through the first phase of our project. The videos were all publicly available on YouTube and Facebook.

For the paediatrician story: Arnaud Gagneur from Sherbrooke University for the French version ( <https://www.youtube.com/watch?v=B_kpGPZeShI> ) and The Children’s Hospital of Philadelphia for the English version (<https://www.youtube.com/watch?v=xNbVjCdvrdA> ).

For the informed decision-making videos featuring parents: Kids Boost Immunity (<https://www.facebook.com/iboostimmunity/posts/1432957773404880/>).

For the mother’s experience with vaccine preventable disease: Immunize Canada (<https://www.youtube.com/watch?v=mWPZGYAdcnc> ).
