# Supplementary material for: Exploring parents’ views of the use of narratives to promote childhood vaccination online
Source: PLoS One. 2023 Jul 19;18(7):e0284107. doi: 10.1371/journal.pone.0284107 (PMC10355395; doi:10.1371/journal.pone.0284107)
Supplement: S2 File — (DOCX) [file pone.0284107.s002.docx]

**Focus Group Guide**

**Introduction (10 minutes)**

- Welcome participants
- Introduce the context of the meeting
- Introduce the meeting: duration, recording, observers, questions and comments, etc.
- Go around the table to invite participants to introduce themselves: first name, region, number of children, profession

- Spontaneously, what comes to mind if I say the words "vaccination OR childhood immunization"?

**(70 minutes) - Discussion of concepts and co-creation of stories**

- In order to answer the various questions parents may have about vaccinating their children, we are looking to develop stories, in the form of testimonials or short stories, and we need your help to ensure that what we develop is useful and relevant. We have some concepts and ideas that we would like to share with you. We would now like to discuss with you three stories we would like to develop about childhood immunization.

First story:

Health Care Professional (Story #1)

**Main questions:**

- What are your first impressions?
  - What are the strong points of this story?
  - Is there enough nuance in what is said about vaccination? (Balance of risks and benefits)
- How could this story be improved?
  - Did you find the story easy to understand?
  - Were you able to understand the key messages in this story?
  - Is there any relevant information we could add that would better address your concerns? (e.g., statistics or reliable sources of information)

**Story-specific questions:**

- How do you feel about having a health care professional tell the story? Would you have preferred another type of health care professional? (Example: nurse, orderly, paramedic, etc.)
- How do you feel about a health care professional telling his story?
- Do you feel that the information provided by the health professional is reliable? (Trust)

Presenting the Informed Decision Story (story #2)

**Key Questions (To be repeated after each story) :**

- What are your first impressions?
  - What are the strengths of this story?
  - Is there enough nuance in what is said about vaccination? (Balance risks and benefits)
- How could this story be improved?
  - Did you find the story easy to understand?
  - Were you able to understand the key messages in this story?
  - Is there any relevant information we could add that would better address your concerns? (e.g., statistics or reliable sources of information)

**Questions specific to the story**

- Does the situation seem believable to you?
- Do you find it easy to seek online information?
- Do you watch videos online? If so, where? (Facebook, Youtube, etc.)

We will now move on to the third story:

Showing anticipated regret (Story #3):

Video by Benjamin

**Main questions:**

- What are your first impressions?

- What are the strengths of this story?

- Is there enough nuance in what is said about vaccination? (Balance of risks and benefits)

- How could this story be improved?

- Did you find the story easy to understand?

- Were you able to understand the key messages in this story?

- Is there any relevant information we could add that would better address your concerns? (e.g., statistics or reliable sources of information)

**Story-specific questions:**

- Do you feel that a story told by a parent is more engaging or interesting than one with a health care provider?

- What do you think of the story? (Touching? Emotional? Manipulative?)

**More general questions following the stories**

- Which story resonated with you the most? Why or why not?

- Which story was least interesting to you?

- Did any of the stories make you feel more equipped to make an informed choice about vaccination? Why or why not?

- What suggestions do you have for the best ways to reach parents like you (written, video, person speaking, tone, length, effects (e.g. cuts with text like the whooping cough story and on what platform? (Facebook, Instagram, TikTok, etc.)?

- Which terms should we add or not present in the video (ex: words like, pain, risks, etc.)

-Which specific information should we add in the video?

-Explore the possibility of a conversion story

- Is there anything else you'd like to share with us about vaccination or the material we discussed today?

**Conclusion (10 minutes)**

- Take note of participants' questions and contact information in order to return a response

- Inform participants of the possibility of being contacted again to submit a more developed concept.

- Discuss the terms and conditions of the compensation

**90 minutes**
