# Supplementary material for: Exploring parents’ views of the use of narratives to promote childhood vaccination online
Source: PLoS One. 2023 Jul 19;18(7):e0284107. doi: 10.1371/journal.pone.0284107 (PMC10355395; doi:10.1371/journal.pone.0284107)
Supplement: S3 File — (DOCX) [file pone.0284107.s003.docx]

**Verbatims**

**Participants’ comments on the video about parents informed decision making**

FG01, 001: Moi je trouve juste ça bizarre qui parle de non-information parce que la première chose que le médecin nous dit quand notre enfant… Même pendant le suivi de grossesse, ils parlent déjà des vaccins, puis dès qu’on a le premier rendez-vous, déjà ils nous en parlent, donc je trouve ça un peu drôle qu’ils disent ça. Mais je ne sais pas si c’est parce que c’est en France ou que ça fonctionne différemment.

FG01, 002 : Oui bien c’est ça, aujourd’hui quand nos enfants naissent, ils nous remettent le carnet vaccinal. Après ça à tous les suivis, je pense qu’ils nous demandaient : « ses vaccins sont-ils à jour? Ses vaccins sont-ils à jour? » Alors, c’est un peu difficile… En tout cas avec le staff qu’on a rencontré, c’était toujours un bon suivi par rapport à ça, ils posaient les questions…

FG01, 003: Bien un peu dans le même sens, je trouve que le vidéo… La première chose je trouvais que c’était un vieux vidéo justement. Je trouvais que même l’ensemble faisait vieux un peu. Mais c’est justement pour avoir passé par là, ils nous donnent même un dépliant maintenant qui décrit tous les vaccins qu’on a à quel âge et tout. Je veux dire c’est tellement bien indiqué. Donc c’est sûr que le vidéo, je trouve qu’il vient moins me rejoindre. Peut-être plus dans la manière qu’il dit qu’il a vit un enfant mourir de ses propres yeux, des fois on dirait que les gens ont besoin d’entendre ça pour comprendre, mais sinon c’est pas mal ça.

FG2

**Participants’ views on the fact that the testimony is from an health care provider**

001: Bien moi je pense que oui. Parce qu’on voyait quand même qu’il a dû en voir plusieurs, puis on voyait que ce cas-là l’avait marqué particulièrement, donc peut-être de voir qu’il n’est pas blasé, puis il fait vraiment ça pour aider les gens, ça peut aider aussi. Tu sais, ce n’est pas juste une maman émotive ou n’importe quoi, c’est quelqu’un quand même d’assez… c’est un scientifique qui a lu, qui a eu des études, puis ça le touche quand même, donc pourquoi on ne l’écouterait pas?

001: Bien je trouve le médecin crédible, mais de mon expérience que j’ai été vraiment beaucoup à l’hôpital, puis les personnes que j’ai le plus vues, c’était les infirmières. Donc oui, peut-être que ça peut plus parler parce que ce sont eux [elles] qui sont plus en contact avec les patients. Tandis que les médecins, bien on les voyait 2 minutes par jour. Puis on a peut-être moins un contact avec eux.

002: Effectivement je trouve que oui ça a de la crédibilité. Mais je suis un peu comme [001], moi je porte plus attention à ce que les infirmières disent. Parce qu’aussi on a eu quelques cas à aller à l’hôpital, et souvent… je ne sais pas les médecins des fois, on dirait qu’ils sont bornés. Tandis que les infirmières sont plus ouvertes. Elles donnent vraiment plus un feedback sur ce qu’elles voient sans avoir peut-être ces œillères-là, alors souvent on apprécie plus ce que les infirmières vont dire. Pas qu’on n’écoute pas le médecin, mais pour nous, les infirmières ont un petit peu plus de crédibilité quand c’est de rapporter des choses qui se passent dans la population.

**Participants’ views on the way that vaccination is described in the video**

003: Bien moi c’est ça, je trouve que même… Il ne faut pas parler de COVID, mais je trouve qu’on n’est pas assez informé non plus des risques de vaccination. On se fait souvent dire:« non, non il n’y a pas de risque, il n’y a pas de risque», mais minimes qu’ils peuvent être, ça peut être le fun aussi de savoir c’est quoi les risques. Je trouve qu’on a tendance à les cacher un peu aussi.

003: Oui. Peu importe que ça puisse être minime ou gros. Même avec mon coco, des fois je n’étais même pas informée des symptômes après le vaccin, c’est par après quand j’appelais, ils me disaient : « ah bien c’est sûr que ça a rapport au vaccin». «-Ah! OK». Donc je trouve qu’on en parle pas assez de ça.

002: Moi, je me souviens qu’ils vont mentionner : «il peut faire un petit peu de fièvre...», tu sais les classiques :« au lieu d’injection, il va y avoir une rougeur», mais ce n’est pas vraiment eux que je trouve qui sont importants, ce sont les risques 1%. Parce que les 1% souvent sont graves, et là tu veux le savoir. Puis s’il y a de quoi, tu veux aller rapidement aller à l’hôpital. Et non pas dire :« ah je vais attendre c’est peut-être...», on ne le sait pas. Alors ça, effectivement, ils n’en parlent pas. Je sais que probablement c’est pour ne pas faire peur au monde. Mais tu sais quand on va chercher un médicament à la pharmacie, ils vont remettre un papier, puis au moins on peut lire, même les risques qui sont rares sont tous là, puis ils sont bien marqués ‘’rares’’ et tout ça. Je ne me souviens pas avoir eu ça pour les vaccins. Je ne crois pas.

003: Peut-être pas dans un vidéo. Comme [002] dit peut-être parce que ça fait peur aux gens, mais peu importe dans un vidéo, quand on parle au médecin, quand on a un dépliant, on a rarement les risques graves. On se fait tout le temps dire qu’il n’y en a pas, qu’il n’y a pas de risque, mais on le sait qu’il y en a, c’est juste qu’ils sont très minimes.

002 : Tu sais, des informations qu’on a toujours demandé quand on a fait vacciner c’était… Puis justement avec le vaccin de la COVID, je pense que c’est d’actualité de dire : « est-ce que c’est un vaccin? Ça fait combien de temps qu’il existe?», donc est-ce que les effets sont connus? Bien on le sait que les anciens vaccins… COVID a été fait rapidement, mais les autres normalement, c’est un 10 ans d’étude bien suivie, bien s’ils pourraient juste nous dire : « ah ce sont des vaccins… ça fait 15 ans, 20 ans, 50 ans qu’ils existent, on les connaît bien». Le fait de bien les connaître, au moins, même s’il y a des complications, ça veut aussi dire qu’ils savent probablement comment réagir à ça. Ça, ce sont des trucs que je trouve qui seraient peut-être intéressants… Le message qui passe par rapport à ça.

001: Moi je suis d’accord justement c’est ça qui me rendait plus hésitante avec le vaccin de la COVID. J’ai toujours fait vacciner, mais on dirait que celui-là me rendant plus hésitante, justement parce que c’est un nouveau, puis qu’on ne sait pas trop. Mais c’est vrai que de savoir que c’est un vaccin que ça fait 20 ans, tu te dis : « OK, ils auraient arrêté de le donner si ça ne marchait pas».

**Histoire #2**

***« Des parents hésitent à faire vacciner leurs enfants puissent qu’ils ont eu de fausses informations sur les vaccins. Ils se sentent submergés par toute l’information disponible en ligne. Ils ne savent pas où trouver les bonnes informations et quelle est la meilleure décision à prendre pour leurs enfants. À force de chercher en ligne sur des sites gouvernementaux et en comparant les informations avec les sites anti-vaccins, ils prennent la décision de faire vacciner leurs enfants».***

**Je vais commencer le vidéo. Le vidéo est en anglais, mais il a des sous-titres en français. J’espère que vous allez voir les sous-titres synchronisés avec le son.**

**Vidéo #2 ([deux parents hésitants])**

**Donc vos premières impressions, côté positif / côté négatif, est-ce que quelqu’un veut se lancer?**

002 : Bien le bout qu’elle a dit la madame que je trouvais intéressante effectivement c’est… Ce serait bien les médecins quand ils parlent des vaccins ou infirmières, ils disent justement on fait vacciner pour la varicelle, si tu ne fais pas vacciner, les chances que ton enfant l’attrape sont de tant, tandis que s’il est vacciné, les chances qu’il ait un effet secondaire sont de tant. Donc «il a une chance sur 20 de l’attraper puis avec des conséquences graves. Ou, de l’autre côté tu as une chance sur 10 000 d’avoir un effet secondaire négatif important», alors ça permet peut-être de juger le risque d’un côté et de l’autre. Ça, je ne l’ai jamais entendu effectivement par rapport à ça. Bien tu sais comme moi j’ai une maladie auto-immune, puis c’est pour ça que les médecins des fois j’ai de la misère avec leur… ils sont stricts. Quand tu demandes:« est-ce que c’est sécuritaire?», puis ils font juste te dire: «oui». Moi, ça ne clique pas. Parce que moi ça a pris 15 ans… ils me disaient que c’était dans ma tête, ça a pris 15 ans avant d’être diagnostiqué. Alors des fois, tu lis sur les vaccins puis tu te dis : « est-ce que ça cause de l’autisme ou pas?». Puis eux autres «-non». Non, ça ne marche pas juste la réponse «non», mais de dire : « écoute si tu ne le fais pas vacciner, il a une chance sure tant qu’il le pogne. D’un autre côté, tu as peut-être une chance sur un million qui ait l’autisme. En fait, on n’est pas capable de faire le lien parce que c’est peut-être trop rare, mais on ne le nie pas». Moi le côté catégorique, souvent c’est ça qui me met off par rapport aux messages qu’ils essaient de passer, parce qu’on sait qu’il n’y a pas d’absolu dans la vie, on ne peut pas être catégorique à savoir si c’est vrai ou pas vrai, mais s’ils nous donnent des chiffres comme ça, je trouverais ça intéressant ce côté-là.

**OK, donc une information plus nuancée… Est-ce qu’appuyer par des données scientifiques, c’est intéressant pour vous? Ou, c’est plus appuyer par des témoignages? Qu’est-ce qui vous rejoint le plus?**

002: Moi c’est sûr que c’est plus le côté scientifique, témoignage c’est souvent plus subjectif. Mais même là, on ne peut jamais vérifier la qualité, mais là il faut se fier justement à ceux qui nous envoient le message.

**Merci. Quelqu’un d’autre veut se lancer?**

003 :Bien j’ai trouvé que c’était la réalité de tout le monde aussi d’aller faire ses recherches, puis de lire des affaires épouvantables, on peut en voir de toutes sortes. Mais des fois, c’est difficile de départager des témoignages qu’on lit sur internet avec les vraies données, donc j’ai trouvé que… Ça venait me rejoindre parce que j’ai fait la même chose pour le COVID, j’ai fait mes recherches, puis j’ai paniqué, puis finalement je me suis informée aux bonnes places, puis finalement j’étais en paix avec ça, mais c’est venu me rejoindre pour ça,

001 : Je pense que c’est vrai ce qu’ils disaient dans le vidéo, c’est plus facile de trouver des informations négatives ; la personne qui a eu l’effet secondaire de 1 sur 10 000, c’est sûr qu’elle va le dire sur Facebook qu’elle a eu ça, mais tout le monde qui a eu l’expérience positive ne le dit pas, donc là tu ce que tu retiens, c’est la personne qui a eu quelque chose de négatif. Donc ça, je pense que c’est bien qu’ils le mentionnent. Mais c’est aussi difficile des fois de savoir où chercher exactement si le gouvernement ne le met pas facilement accessible, tu ne le trouves pas.

002 : Souvent c’est ça, on va trouver sur un site gouvernemental, ça va dire : « le vaccin est sûr et sécuritaire», rien d’autre. Tandis que sur le site de désinformation, il y en a… puis là, ils vont aller chercher des témoignages, mais tu ne le sais pas ça, est-ce que c’est vraiment relié? S’il n’avait pas été vacciné, est-ce qu’il aurait eu la même chose? C’est ça qu’on… Mais ils en mettent du texte. Tandis que de l’autre côté, ils en mettent à peine puis, puis encore une fois c’est «oui, c’est sécuritaire», puis ça fini là. L’autre chose que j’aimerais ça… Je me souviens, quand j’étais petit j’ai été vacciné varicelle et rougeole, je crois, les deux, mais on l’attrapait pareil. On l’attrapait moins fort. Tandis qu’aujourd’hui les enfants ne l’attrapent plus. Bien c’est là que j’ai appris qu’il y avait deux catégories de vaccins, celui qui vraiment empêche le virus de rentrer dans ton corps, et l’autre qui fait juste traiter les symptômes. Puis c’est là que j’ai appris que le vaccin avait évolué avec le temps pour ceux-là. Ça aussi c’est de l’info que je trouverais intéressante, c’est de dire : « ce vaccin-là empêche le virus», puis là sûrement les parents diraient : «oh, intéressant». L’autre atténue les symptômes, aussi ça peut être intéressant. Mais on n’a pas… Je ne savais même pas qu’il y avait deux types de vaccins comme ça, avant la COVID, c’est avec la COVID que je l’ai appris. Puis ça m’a fait penser justement à la rougeole et tout ça, puis je posais des questions à ma médecin de famille, puis elle a dit : «oui, oui, oui ceux-là à cette heure c’est rendu qu’ils empêchent le virus d’entrer dans les cellules». On dirait que des fois, ils cherchent trop à être maternel, à ne pas donner d’info plus poussées, en se disant:« ils ne comprendront pas», quand tu fais ça effectivement le monde commence à perdre confiance.

**Est-ce que des informations sur l’efficacité du vaccin, par exemple, si une personne s’en va en contact avec la rougeole, le vaccin est efficace à combien…. À quel point le vaccin nous empêche de l’attraper, par exemple? Est-ce que des informations par rapport à l’efficacité, si vous êtes en contact, est-ce que c’est quelque chose d’intéressant aussi?**

003 : Bien oui.

002 : Justement dernièrement je lisais sur le vaccin contre le paludisme qu’il y avait en Afrique. Ils disaient qu’il était juste 40%, mais pour eux autres c’était quand même mieux que rien, ça sauvait quand même 200 000 personnes. Je trouvais que c’était : «ah, c’est juste 40%» «- oui, mais on sauve 200 000 enfants, pareil. Il y a 200 000 morts de moins». «- OK, c’est quand même…». Donc des chiffres comme ça, ça peut être intéressant, puis même si le pourcentage est bas, d’expliquer la différence avant le vaccin, puis après le vaccin.

001 : Bien c’est vrai, je pense que ça aiderait peut-être de dire justement parce que là on a l’impression que c’est juste protéger nos enfants, mais on oublie les autres enfants. Comme admettons quand mon fils était immunosupprimé, bien il n’est pas le seul, il y a plein de maladies auto-immunes, tu t’en vas à l’école, puis tu n’es pas protégé parce que les autres ne l’ont pas, mais toi ce n’est pas par choix, c’est parce que tu ne peux juste pas le recevoir, puis je trouve qu’il manque un peu ça aussi dans le discours. Est-ce qu’on peut y aller plus en société que juste protéger ton enfant? Bien c’est que tu protèges d’autres enfants d’autres parents qui sont inquiets parce que leurs enfants sont laissés un peu à eux-mêmes, ils ont des traitements, ils s’en vont à l’école. Je ne sais pas si ça aiderait.

**Donc de parler un peu de protection communautaire, puis d’expliquer le principe.**

001 : Oui, bien c’est parce qu’on ne le savait pas. Avant que mon enfant soit malade, je n’y pensais pas

à ça, puis c’est normal. C’est juste que je pense que ça aiderait peut-être de dire : « oui c’est vrai, je ne pensais pas à ça qu’il y en avait d’autre, mais oui...». Il aurait pu en mourir de la varicelle admettons pendant… Donc là tu te dis : « OK, ce n’est pas juste...». Moi je pensais que c’était juste des petits boutons qu’on grattait. Quand tu vois que ça va au-delà de ça…

**Est-ce que vous avez d’autres choses à mentionner sur ce récit-là? De ce que je comprends, c’est que vous vous reconnaissez vraiment la recherche chez ces deux parents-là, de rechercher de l’information, puis d’essayer de trouver les informations que vous voulez par rapport aux vaccins, que ça soit difficile sur internet de savoir où chercher. Est-ce que pour vous quand vous arrivez sur un site gouvernemental, par exemple, est-ce que l’information… même si, de ce que je comprends, c’est qu’elle n’est pas nécessairement complète, mais est-ce que l’information présentée vous faites confiance à l’information gouvernementale ou est-ce qu’il y a d’autres sources que vous faites davantage confiance?**

002 : En fait, moi je trouve que l’information est tellement petite, il n’y en a pas. «Tel vaccin, c’est de 3 à 1, il est sécuritaire», puis ça finit là, il n’y a rien d’autre. Alors je ne peux pas dire, est-ce qu’elle est bonne? Est-ce qu’elle n’est pas bonne? Je trouve qu’il n’y en a pas.

003 : Je sais que ce n’est pas le même groupe, mais moi j’ai trouvé un groupe de femmes enceintes vaccinées, puis ça m’a tellement donné aussi des bonnes informations parce que c’était toutes des femmes enceintes vaccinées qui donnaient leurs informations. Puis d’avoir trouvé ça, ça m’a tellement fait du bien à comparer admettons à un site du gouvernement, parce que là j’avais des témoignages positifs. Donc c’est sûr que d’avoir des témoignages positifs, je pense que ça vient plus me chercher que d’aller voir sur le site du gouvernement.

002 : Ou, encore, les vaccins ils vont dire « de 3 à 1...», mais après ça tu te dis : «bien je vais peut-être aller sur le site...». Parce que les sites aussi qui ont beaucoup d’info, ce sont ceux qui manufacturent, eux autres ils en ont vraiment beaucoup, mais tu ne le sais pas c’est qui qui l’a manufacturé, ils ne donnent pas non plus cette info-là. Peut-être parce que ça change selon les appels d’offre ou autre, mais ça serait bien de dire : «regarde, les vaccins cette année, c’est ça, si tu veux de l’info, un lien vers celui du manufacturier», puis eux autres souvent ils te listent vraiment tout tout tout tout parce que c’est l’info qu’ils rendent publique… Bien qu’ils ont aussi donné aux différents gouvernements pour faire homologuer. Ça, on ne l’a pas, alors tu te dis : « je vais aller sur lui, mais est-ce que c’est vraiment Merck qui a fait ce vaccin-là?», je ne le sais pas.

003 : C’est vrai ça qu’on ne le sait pas, puis que c’est dur à chercher.

**Oui, c’est vrai que c’est assez compliqué si on veut toutes les informations super pointues, effectivement les monographies de produits, c’est tout écrit, mais c’est de retrouver quel vaccin vont être administré, puis qui a fait le produit, puis sur quel site chercher, puis si c’est en français, des fois ce n’est même pas en français. Donc effectivement ce sont des informations difficiles à trouver. Vous avez dit aussi un petit peu [003] que vous étiez sur les médias sociaux, dans le fond vous avez un forum de mamans qui vous a aidé, qui vous a rassuré par rapport à des décisions de santé, est-ce que vous regardez des vidéos en ligne aussi? Là, je m’adresse à tout le monde, est-ce que pour vous, vous regardez par exemple sur Facebook des vidéos en ligne ou YouTube? C’est quoi les plateformes de vidéo que vous regardez?**

003 : Bien moi dans ce groupe-là, justement il y a beaucoup de femmes qui mettent des liens. Ce sont des vidéos qui viennent de la France, mais je crois que ce sont des liens YouTube qu’ils mettent. Des vidéos de médecins de la France et tout, là.

002 : Dans mon cas, non, pas de vidéo, ni YouTube ou Facebook.

001 : Moi non plus.

**Donc ce serait sur quelle plateforme par exemple, si on faisait avec un témoignage? Je ne sais pas si vous allez chercher ce genre de vidéo là pour prendre des décisions, mais si jamais vous n’allez pas chercher ces vidéos-là, mais que vous aimeriez le regarder… de quelle façon vous aimeriez recevoir ce genre de vidéo là?**

002 : Bien en fait si c’est le site gouvernemental, puis qu’ils veulent passer un message puis qu’ils mettent la vidéo imbriquée dans le texte, je vais le regarder. Si ça me tente d’aller chercher ce type d’information-là, mais sans ça… Aller sur YouTube pour faire une recherche là-dessus, je n’y pense… non, ce n’est pas le style. Je vais aller voir de la musique. Mais tu sais de me dire : « ah un message gouvernemental, va là-dessus», je n’y penserais même pas.

003 : Non, puis même que je viens de me rappeler, je pense que le lien ce n’était même pas YouTube, c’était un lien qui allait sur un hôpital de la France, donc c’était vraiment directement. Donc c’est sûr que sur les sites d’hôpitaux, sur le gouvernement, peut-être que j’irais plus chercher là, oui.

001 : Sainte-Justine, peut-être.

003 : Oui, Sainte-Justine.

001 : Si on a une question, c’est quand même là que tout se passe. Au Children.

**Puis admettons que le vidéo serait sur la page Facebook de Sainte-Justine, par exemple, est-ce que c’est du contenu que vous feriez confiance?**

001 : Moi, oui.

003 : Moi, aussi.

002 : Oui, ça vient quand même une source officielle. Sur un site comme ça, pour les nouvelles, tu te dis : « c’est eux autres qui contrôlent cette page-là». Alors, oui.

**Est-ce que vous aviez d’autres choses à ajouter par rapport à ce témoignage-là de parents qui avaient de la difficulté à trouver de l’information en ligne?**

[Les participants font le signe ‘’non’’]

**On a fait le tour. Donc là, je vais vous présenter le troisième témoignage. Je vais juste vous avertir que celui-là est très touchant parce que c’est une maman qui a vécu le fait que son fils n’était pas vacciné et maintenant il a un handicap, donc l’enfant est dans le vidéo puis elle témoigne de ça. Donc c’est très touchant, la première fois que je l’ai regardé, j’ai eu des larmes aux yeux. Donc je voulais juste vous avertir, puis vous préparer psychologiquement. C’est aussi un vidéo qu’on retrouve en ligne, qui est disponible, puis c’est un vidéo en français aussi.**

**Histoire #3**

***« Une mère témoigne des complications que son enfant a eues à cause d’une maladie évitable par la vaccination. Elle ne savait pas que le vaccin existait et parle de son expérience à l’hôpital et de l’impact sur sa vie actuelle avec un fils handicapé ».***

**Lecture du vidéo 3** (témoignage maman et enfant)

**Donc, vos premières impressions? Points forts / points faibles? Est-ce que vous avez aimé ce type de vidéo?**

002 : Bien moi je trouve que c’est important justement de rappeler ce que les vaccins amènent parce qu’on ne les voit plus souvent ces maladies-là. Alors de rappeler et de dire : « le taux de mortalité infantile avant les vaccins étaient très élevés, puis ça aide...». Mon frère a fait une méningite bactérienne au lieu d’être virale, et il a quand même failli en mourir, mais celle-là au moins elle n’est pas critique comme la virale. Donc oui, il faut les rappeler. Puis la médecine moderne a des défauts, mais elle a des qualités, puis ça, ça fait partie des qualités, c’est-à-dire que le taux de mortalité de nos enfants… Tu sais je fais juste regarder mes deux enfants, les deux se sont ramassés à l’hôpital avec des pneumonies incroyables en étant en bas âge, puis si on n’avait pas ce qu’on avait aujourd’hui, probablement qu’ils ne seraient pas là aujourd’hui, ils n’auraient pas passé à travers. Alors, oui, je trouve que c’est un bon cas, nous rappeler que ces maladies-là infantiles, ce n’est pas parce qu’on ne les voit plus ou pas beaucoup qu’elles n’existent plus, elles sont vraiment là. Puis que si on arrête les vaccins, bien justement, elles vont probablement revenir avec les générations.

**Merci. Est-ce que quelqu’un d’autre a quelque chose à dire?**

001 : Je suis d’accord avec ce que [002] dit, c’est vrai qu’on a l’impression que les maladies n’existent plus. On se dit : « ah on n’a plus besoin de vacciner parce que ça n’existe plus», puis là le monde arrêtait de se faire vacciner, puis là on voit que admettons les oreillons sont revenus, puis ça c’était disparu, donc je pense que c’est important de voir quand même ce que ça peut faire. C’est tout.

003 : Bien oui je suis d’accord aussi avec ça. Puis je trouve qu’un vidéo d’impact comme ça, je trouve que moi ça vient me chercher beaucoup. Donc je trouve ça bien comme vidéo. Mais moi j’avais juste une question, elle dit qu’il n’était pas disponible le vaccin?

**Pas au moment que son fils était jeune, c’est quand même un vidéo qui n’est pas très récent.**

003 : Ah OK, c’est assez vieux, c’est pour ça que je ne comprenais pas.

**Vu que c’est comme un vidéo… Je pense que c’est le vaccin qui est donné à 12 mois dans le calendrier vaccinal. Si c’est un autre type de méningite, c’est le genre de méningite qu’il y a un cas dans une région, on vaccine toute la région au complet par prévention, donc c’est vraiment très rare que quelqu’un va attraper ce type de méningite aujourd’hui. Mais à l’époque c’était plus fréquent, donc c’est surtout ça.**

003 : OK.

**Donc de ce que je comprends, c’est que le témoignage par exemple d’une mère avec son fils, c’est quelque chose qui vient vous interpeler, si je comprends bien?**

003 : Oui.

002 : Que ce soit la mère ou un des parents, n’importe lequel.

**Parfait, donc mère ou père, ou quelqu’un de proche. Est-ce que vous aimez mieux un récit avec un parent ou avec un professionnel de la santé? Est-ce qu’il y en a un qui vient plus vous chercher que l’autre?**

001 : Bien c’est sûr : le parent. Parce que lui est plus émotif, mais peut-être balancer avec un professionnel de la santé aussi pour pas que ça vire juste dans le drame.

003 : Moi, je ne pourrais pas dire, les deux je trouve qu’ils viennent me chercher quand même. Je ne sais pas lequel a le plus d’impact.

**Puis [002] est-ce que vous aviez une opinion par rapport à ça?**

002 : Bien, je me posais la question. Je pense que c’est plus le parent… L’autre je ne le mets pas de côté non plus, mais on est parent, alors on a de l’empathie, je pense, plus pour le parent qui donne son témoignage que pour le médecin. Mais le médecin a quand même la crédibilité.

**Ce type de témoignage là, est-ce que vous avez l’impression que ça joue sur les émotions? Si oui, est-ce que c’est positif ou négatif? Est-ce que ça joue un peu trop? Est-ce que vous vous sentez un peu manipulé? Pouvez-vous me donner un peu votre avis là-dessus?**

003 : Ça doit dépendre du style de témoignage, mais je trouvais qu’elle [participant fait référence au vidéo 3 – maman + enfant] ça avait l’air vraiment senti quand elle parlait ça avait l’air vraiment… Je n’ai pas trouvé que ça avait l’air du chantage et tout, ça doit dépendre de la personne qui fait le témoignage, mais celui-là vient nous chercher.

002 : Je pense que… comme elle [participant fait référence au vidéo 3 – maman + enfant] on sentait que c’était vrai, on sentait l’émotion qui sortait. Disons que s’ils essayaient de prendre un acteur pas connu, là tu te dirais:« ah, non, là ils essayent», puis là ça ferait l’inverse. Là je pense qu’on sentait… Puis en ayant son enfant à côté, il est toujours vivant tant mieux malgré tout ce qu’il lui est arrivé, mais on le voyait, c’était son enfant. Donc il n’y avait pas de fake, rien. Ça fait mieux passer le message, puis je ne trouvais pas que c’était trop.

**Merci [001] est-ce que vous avez quelque chose à dire?**

001 : Non. Bien moi je suis d’accord. Je dis juste le professionnel de la santé… C’est juste que moi je suis bien sensible, puis ça me fait toujours pleurer, donc je me dis que ça me tempérait un petit peu. Mais non, c’était un bon vidéo. Parce que j’ai l’impression que les gens quand ce sont des médecins, ils ne leur font pas toujours confiance non plus, ceux qui sont contre la vaccination, mais de voir ce que ça peut faire, tu peux te dire: «ouais c’est vrai… je ne pensais pas à ça, puis finalement je le vois, puis je n’aimerais pas ça que ça arrive à mon enfant».

**Est-ce qu’il y a un type de maladie qui est évitable par la vaccination que vous pensez qui serait plus important de parler dans un vidéo, par exemple?**

001 : Bien j’avais entendu dire que la coqueluche était revenue chez les nouveau-nés, donc ça peut-être. Parce que moi, on m’en a juste parlé quand j’étais enceinte, puis je ne le savais pas. Donc peut-être ce qui est d’actualité en ce moment, ça pourrait être intéressant.

**C’est vrai que la coqueluche est d’actualité en ce moment. C’est pour ça que maintenant on donne le vaccin aux femmes enceintes pour pouvoir donner des anticorps aux bébés qui naissent. Donc c’est vrai que ça serait quand même d’actualité. Sinon, je pense que la rougeole est aussi un petit peu d’actualité, c’est revenu, il y a des éclosions parfois. Donc est-ce qu’il y a une des maladies… laquelle vous interpelle le plus? On va y aller avec [002].**

002 : Je ne le sais pas parce que justement, là on parle de la coqueluche, oui je connais la coqueluche de nom, mais c’est quoique ça donne? Même la rougeole, la varicelle, je les ai pognés, mais moi c’était on grattait parce que j’avais été vacciné… on prenait un bain avec de la petite vache, mais on ne sait plus qu’est-ce qu’elles font ces maladies-là. On ne le sait plus, donc je ne pourrais pas dire. Bien la méningite, ça je pense qu’on la connaît tous, quand tu l’attrapes les chances d’y rester sont très élevées. Mais le reste… Comme les oreillons, on m’a dit que si tu pognes ça adulte, peut-être… infertilité. Puis même ça, c’est disparu beaucoup. Alors je ne pourrais pas dire, pourtant je me tiens informé, mais les maladies infantiles, je ne me souviens même pas qu’est-ce qu’elles font en bout de ligne, si elles ne sont pas traitées… Je ne le sais pas.

**Donc peut-être d’inclure ces informations-là quand quelqu’un témoigne. Tu sais comme ça a été fait dans le vidéo de la méningite, on a vu les complications de la maladie, donc c’est quelque chose qui serait intéressant de mettre dans un récit ou un témoignage vidéo?**

002 : Oui.

003 : Oui, parce que moi aussi je n’en ai aucune idée. On connaît les noms, mais on n’a aucune idée de ce que ça peut faire.

002 : On connaît les noms et quelques symptômes, mais jusqu’où ça va ces maladies-là si elles ne sont pas traitées, s’il n’y a pas eu de vaccin, je pense qu’il y a plus grand monde qui le savent.

**Parfait. Maintenant on va faire des questions plus générales sur les trois vidéos ensemble. Donc j’aimerais savoir lequel vous a le plus interpelé et pourquoi? Donc pourrait commencer avec [001].**

001 : Euh, bien je pense que c’est le troisième qui m’a le plus interpelé. Bien justement parce qu’on voyait l’enfant peut-être, ce n’était pas juste théorique, c’était vraiment concret. Mais en même temps, j’ajouterais peut-être plus des données scientifiques comme quoi… Admettons plus de pourcentage, c’est quoi les chances? Parce que oui, je pense que ça peut aussi aider les gens. Parce qu’on a toujours l’impression que ça ne nous arrivera jamais, mais de le voir en chiffre admettons 1 sur 300, bien c’est quand même beaucoup. Des choses comme ça.

**Donc le pourcentage de chance de l’attraper, si on ne se fait pas vacciner?**

001 : Bien de l’attraper ou des complications. Juste ajouter un élément plus d’information admettons avec le témoignage émotif aussi du parent.

**Parfait. Puis d’ajouter aussi par exemple des informations sur l’efficacité du vaccin, en plus, ou quelque chose comme ça?**

001 : Oui, ça, ça pourrait être intéressant. C’est vrai qu’on ne le sait jamais vraiment.

002 : Bien moi, c’était 3-2-1 dans l’ordre. Le troisième oui, il vient nous chercher, c’est un témoignage direct. Le deuxième, ce sont des questions qu’on se pose tous, alors c’était très d’actualité. Le premier, ce n’est pas qu’il était mauvais, mais il venait moins me chercher que les deux autres. Puis tu sais aujourd’hui avec internet, l’information est partout, mais on ne sait pas si c’est la bonne, si ce n’est pas la bonne? Alors le deuxième venait me chercher de ce côté-là. À un moment donné, il y en a trop d’information. À l’époque c’était l’inverse, notre seule source d’information, c’était le médecin qui des fois… on a tout le temps l’impression de les déranger. Alors je trouve que c’est bien aujourd’hui, il y a le plus et le contre. Donc le témoignage de la madame avec son enfant à côté, c’était le mieux.

003 : Bien moi, c’est le deuxième aussi qui vient me… Je me reconnais vraiment là-dedans. Depuis les derniers mois, j’ai beau poser des questions à mon médecin sur la vaccination, toutes ses réponses sont : oui, non. Mais je n’ai pas plus d’information. Donc c’est moi qui vais faire mes propres recherches, puis je me perds un peu là-dedans. Donc c’est sûr que le deuxième vidéo est venu me chercher un peu plus.

**Puis juste me mentionner, tout le monde, quel vidéo… bien [002] vous l’avez mentionné, le premier vidéo vous interpelait moins. Mais [001] et [002], lequel des vidéos vous interpelait le moins? On peut y aller avec [001] en premier.**

001 : C’est le premier qui m’interpelait le moins.

002 : Oui, moi aussi, c’était le premier. Peut être juste aussi dans la manière qu’il a été fait, mais moi aussi, c’est le premier.

**Est-ce qu’il y a un des récits qui vous a fait sentir un peu plus outillé pour prendre une décision de vaccination? Est-ce qu’il y aurait un vidéo qui aiderait vous ou des parents à prendre une meilleure décision pour leurs enfants, par rapport à ça?**

001 : Le deuxième, je pense. Parce qu’on avait l’impression qu’on n’avait pas à se sentir coupable de se poser des questions, que ce n’était pas parce qu’on pensait ne pas vacciner nos enfants qu’on était nécessairement des monstres. C’est juste que c’est vrai que ça se peut que tu sois mal informé ou que tu veuilles attendre d’en savoir plus avant de décider ça.

002 : C’est pas mal ça. Dans le sens, oui, ça rejoint beaucoup ce que [001] a dit.

003 : Moi c’est un ensemble des trois, je pense, qui fait que ça m’informe plus.

**Puis pour la façon de transmettre un message, est-ce que vous un vidéo, ça vous rejoint? Sinon, est-ce que par exemple un récit écrit ou une bande dessinée…? Le médium pour faire passer le message, est-ce que vous avez une préférence?**

002 : Moi je trouve qu’un vidéo, c’est une bonne introduction, mais avoir peut-être un lien ou une chose pour dire : «tu en veux plus...?» Parce qu’un vidéo, ça reste court, donc c’est sûr que ça ne répond pas à tes questions. Bien, de dire : « tu peux aller à tel endroit pour avoir de l’information de qualité».

003 : Oui, c’est vrai qu’un mélange. Parce que des fois, même un vidéo trop long, j’en ai écouté des fois, c’est trop long que tu perdes l’attention, puis tu le fermes. Donc peut-être un mélange de vidéo et de lien, ça pourrait être bien ça.

002 : Parce qu’effectivement, un vidéo comme [003] dit qui est très long...Tu cherches une info, puis supposons que c’est 1 heure et demie, parce que c’est une conférence, tu ne l’écouteras pas. Tandis qu’un texte avec des hyperliens en haut que tu peux cliquer sur différences sections qui t’intéresses vraiment, c’est beaucoup plus rapide. Alors le vidéo peut être très court, mais dirigé vers une autre place, c’est une bonne combinaison, je trouve. La bande dessinée aussi peut être intéressante, mais plus pour les enfants. Mais là, normalement, ils sont vaccinés quand?

**Parfait, est-ce que quelqu’un d’autre avait un avis sur..?**

001 : Moi j’aime bien lire, donc c’est vrai que ça me rejoindrait plus d’avoir un article à lire. C’est vrai qu’il pourrait avoir un petit vidéo dedans, mais j’aime ça quand il y a des photos, puis que ça explique un peu… Puis chercher les mots-clés, c’est vrai que c’est pratique aussi.

**Pour trouver de l’information?**

001 : Oui.

002 : Souvent le vidéo va passer, puis tu vas l’oublier tandis l’autre [article], tu peux l’archiver garder un bookmark ou le faire imprimer et y revenir. Tandis que le vidéo, même des fois juste le retrouver, ça va être difficile. Parce que chercher un vidéo, n’importe où, c’est presque impossible encore.

**C’est vrai, bon point, je n’y avais pas pensé. Donc vous avez mentionné un peu la longueur du vidéo, pour vous est-ce que ce qu’on vous a présenté c’était trop long? C’est à peu près 3 minutes, les vidéos, c’était trop long? Trop court? Qu’est-ce que vous en pensez?**

003 : C’était parfait.

002 : Il y a juste le premier vidéo, vu que c’est un médecin avec un ton un petit peu plus monotone, il me paraissait un petit peu plus long que les deux autres qui étaient plus animés.

**OK, mais pourtant ils sont à peu près de la même longueur. Mais c’est vrai que des fois quand le vidéo est moins intéressant, on a l’impression que c’est plus long.**

003 : Dans le premier, peut-être vu qu’il était toujours dans le même décor, ça fait plus long, mais les autres on voyait les enfants jouer, on voyait la mère faire différent… Il y avait différents points de vue, donc peut-être que ça passe mieux comme ça. Tu restes concentré puis tu le regardes.

**Puis est-ce qu’il y a des mots qu’on ne devrait pas utiliser quand on parle de vaccination, vous pensez? Des mots qui pourraient faire que des parents plus hésitants, par exemple, voudraient ne rien savoir du vidéo. Des fois il y a des gens qui n’aiment pas ça utiliser le mot ‘’risque’’ ou des choses avec une connotation quelconque. Est-ce que vous pensez qu’il y a des mots qui vous viennent en tête qu’on ne devrait pas utiliser?**

002 : Moi je pense qu’avec les années à force d’essayer d’éviter des mots comme ça, ça ne mène nulle part. Il va toujours y avoir du monde qui va se sentir offenser par un mot ou l’autre. On ne peut pas s’en sortir, tout ce qu’on fait, c’est de diluer tout le temps le message qu’on essaie de passer, puis c’est rendu fou aujourd’hui. Alors je ne pense pas...Passons le message, puis il va toujours en avoir qui vont être contre, qui vont être pour, qui vont dire : «regardez il a dit ça, il essaie de nous faire peur». Plus on enlève des mots de notre vocabulaire, plus les petits mots qui étaient bénins il y a 10 ans, deviennent des gros mots d’aujourd’hui. On ne sent sort pas.

001 : J’éviterais peut-être juste d’essayer de donner des ordres, donc évitez l’impératif, peut-être. Parce que ça, j’ai l’impression qu’il y a des gens qui ne répondent vraiment pas à ça, d’avoir l’impression d’être forcé. Mais sinon le reste... les mots je pense qu’on peut les utiliser sans problème.

**[003] est-ce que vous avez une opinion par rapport à ça?**

003 : Bien ça allait dans le même sens, puis que ce n’est pas trop compliqué aussi. Tu sais des fois, tu entends des mots que tu te dis : « mon dieu qu’est-ce que c’est ça?» Puis de dire les vrais mots.

002 : Effectivement, on pourrait utiliser des mots courant et non pas que tu as trouvé après 7 ans d’université dans les livres scientifiques. Parce que ça, ça fait plus ‘’péteux de broue’’ disons. Là, ça ne nous rejoint plus.

003 : Oui, c’est ça. Puis tu perds des gens, puis ça fait : « mon Dieu, ils ne voulaient pas que l’on comprenne que c’était juste ça, finalement».

**Donc, de bien vulgariser les propos quand on parle d’information sur les vaccins, si je comprends bien?**

003 : Oui.

**J’aurais une dernière question pour vous. Si par exemple on explorait l’idée d’un témoignage d’une personne qui était contre la vaccination, puis qui pour une raison quelconque décide d’être pour la vaccination, est-ce que pour vous ça serait quelque chose de plausible? Ou, quelque chose que vous vous reconnaîtriez là-dedans? Par exemple, une mère qui ne voulait pas faire vacciner contre la coqueluche et qui décide soudainement… peut-être parce que son enfant attrape la coqueluche de devenir pro vaccination et de témoigner en fonction d’être pour le vaccin, par exemple?**

003 : Dans le fond tu veux savoir si aimerait ça voir ce genre de vidéo-là?

**Si ça vous interpellerait de voir que quelqu’un était contre, puis qu’ensuite elle est devenue pour ou pro vaccination.**

003 : Je pense que ça dépend vraiment. Bien moi oui, ça viendrait me chercher. C’est sûr que j’aurais un intérêt, ça dépendrait encore du pourquoi, puis de l’intérêt de la personne.

001 : Moi j’aimerais voir son raisonnement, le parcours, pour quoi elle a changé? Qu’est-ce qui l’a fait changer? Pas juste parce que son enfant a été malade. J’aimerais justement une autre raison que ça. Comme de voir… Parce qu’on dirait que c’est trop facile, mais je ne le sais pas.

002 : Moi ça ne vient pas me chercher du tout parce que c’est toujours… Je me dis tout le temps : «bon, ça lui est arrivé à cette personne-là, donc elle change d’idée...», mais on est tous comme ça en tant qu’être humain. Au-delà de la vaccination, c’est… «ah ça m’est arrivé donc là je vais lancer un discours pour ça», oui, mais attends avant que ça t’arrive tu étais de l’autre côté. Il faut comprendre qu’il y a du monde... dans plein de sujets, on a nos opinions et tout ça… Non, ça ne vient pas me chercher du tout, même ça me fait l’inverse. Non, pas du tout.

**Parfait, est-ce que quelqu’un d’autre avait une opinion là-dessus?**

[ Les participants répondent en faisant le signe ‘’non’’ de la tête].

**Donc moi, ça fait le tour de mes questions, est-ce qu’il y a quelque chose qu’on n’a pas dit que vous aimeriez parler par rapport aux vidéos, ce soir, puis les récits qu’on a vu... que je n’ai pas pensé?**

002 : Juste pour le dernier point. Je cherchais un exemple pendant que je parlais. Tu sais un peu comme la vitesse au volant, à un moment donné tu vois un jeune, puis «mon ami est mort, c’est moi qui étais au volant, il faut arrêter ça», mais depuis que je suis tout petit que j’en vois, ça ne marche pas. Ça ne marche vraiment pas.

**Comme si, il était trop tard, une fois que tu changes d’idée.**

002 : C’est ça, c’est toujours après le drame qu’il soit arrivé. Peu importe le drame, il y a du monde qui change d’idée, oui, mais ça ne fera pas changer d’idée celui qui est encore *mindé* comme ça. Ou, que ça ne le rejoint pas du tout. «Ah, c’est arrivé à toi, toi, tu n’étais pas bon...».

001 : C’est ça parce que ça arrive toujours aux autres, c’est pour ça, donc ça ne t’arrivera à toi, même si c’est arrivé à lui, ça ne t’arrivera à toi, donc tu ne changes pas d’idée.

003 : C’est vrai que ces vidéos-là ça vient nous faire poser des questions sur la seconde même qu’on voit le vidéo, mais à long terme, je ne pense pas que ça l’ait d’impact. C’est vrai qu’à long terme… Tu sais sur le coup tu te dis:« ah oui, il a changé d’idée, pourquoi?», mais après ça? C’est vrai que ce ne sont pas des vidéos qui font réfléchir à plus loin.

**Donc c’est super. Bien pour moi j’ai une bonne idée de qu’est-ce qu’on peut ajouter dans les vidéos, comment on peut modifier des choses. Créer quelque chose qui aurait un impact pour des personnes comme vous.**

002 : Juste un mini commentaire. Dans des vidéos médicaux que j’ai déjà vus dans le passé, moi une chose qui ne marche pas du tout, c’est d’entendre une voix qui parlait justement des vaccins, mais tu ne vois pas le visage, moi, ça ne me rejoins pas du tout, du tout, du tout. Ça fait vraiment pub.

003 : Comme les vidéos du gouvernement, souvent. (rire)

002 : Oui, c’est ça. C’est très impersonnel, puis ça ne me rejoint pas du tout. C’est sec. Message de propagande…

**Puis quand on met par exemple des graphiques ou des tableaux avec des statistiques, puis qu’on les présente dans un vidéo, est-ce que ça, c’est quelque chose de…? Puis que quelqu’un parle derrière pour expliquer, mais que ça soit la personne qui témoigne aussi entre, est-ce que c’est quelque chose qui est correct aussi, pour vous?**

002 : Tant que ce n’est pas sec. Justement, moi, voir le médecin, voir les deux parents, voir la mère, ça, ça me parle. Mais supposons que tu n’aurais pas vu le médecin, tu aurais juste entendu sa voix avec des graphiques, zéro. Même si ça avait été le même texte. Parce que je n’ai pas l’impression… Est-ce que c’est un vrai médecin ou c’est un annonceur qu’on a pris pour sa voix? Puis il lit un texte, puis pour lui ça n’a aucun rapport ce qu’il est en train de lire. Puis pour les graphiques, bien c’est sûr que si c’est un vidéo, il ne faut pas qu’il soit trop chargé.

**Est-ce qu’il y avait d’autres choses que vous pensez comme ça qu’on a oublié de mentionner?**

003 : Non je pense que ça décrit bien.

**Parfait. Donc vous aviez quand même des opinions similaires, c’est plus facile dans ce temps-là. Bien merci beaucoup. Puis, s’il y a quoi que ce soit, juste à m’envoyer un courriel, puis demain vous allez recevoir vos compensations.**

003 : Parfait, merci à toi.

Au revoir, bonne soirée!

**Focus group 2**

**Donc spontanément quand je dis le mot ‘’vaccination des enfants’’, j’aimerais savoir ce que vous pensez à premier vue, des vaccins en général. Ce soir on ne parlera pas des vaccins de la COVID parce que c’est un autre sujet un peu controversé, mais les autres vaccins que celui de la COVID pour l’instant qu’est-ce que vous en pensez? Donc, on va commencer par [001].**

001 : Je ne m’attendais pas à être le premier. Moi, comment je vois les choses, c’est qu’on doit laisser une substance(2:41) aux enfants de développer leur propre défense.

002 : Moi de mon côté, c’est un peu l’inverse, je suis très vaccination. Bien je me dis si c’est là c’est parce que ça a été étudié depuis longtemps, puis que ça peut juste optimiser puis aider pour certains virus qui se promènent. C’est sûr, est-ce que… J’ai des inquiétudes parfois, oui. Mais il reste quand même que je fais confiance à la médecine de ce côté-là.

003 : Moi je suis pour les vaccinations oui et non. Oui, je suis d’accord que les enfants s’échangent leurs microbes. Puis des affaires, même les échanges de microbes, bien écoute je ne suis pas spécialiste, mais ce n’est comme pas assez, c’est sûr qu’une vaccination vient protéger. Donc je ne suis pas contre, je ne suis pas pour, la vaccination.

004 : Moi je suis très pro libre choix maintenant, je pense que c’est le choix des parents comme je vous le dis. Personnellement je suis pour faire vacciner mon enfant. Je pense que certaines maladies peuvent être très dangereuses, qu’on peut éviter. Mais encore là, je pense que ça prend l’avis d’un professionnel de la santé pour chacun des vaccins. Je pense qu’il n’y a pas grand parents qui ont une expertise dans ce domaine-là, à part les infirmières. Donc je pense que c’est important [INAUDIBLE4min17]professionnels de la santé.

**Donc maintenant, je vais vous présenter les 3 scénarios de témoignage sur la vaccination des enfants. Moi ce que je vais vouloir savoir par la suite, c’est vraiment votre opinion à vous sur les vidéos parce que dans le fond ce sont des vidéos qu’on voudrait créer qui sont à peu près des scénarios similaires. Donc c’est vraiment un exemple, puis on veut vraiment votre opinion à vous, il n’y a pas de mauvaise réponse. On veut améliorer ça, c’est vraiment comme un genre de brouillon qu’on a en ce moment. Puis c’est sûr qu’il y a des vidéos que vous allez aimer, d’autres que vous n’allez pas aimer, puis ne vous gêner pas du tout pour nous donner votre opinion là-dessus.**

**Donc je vais vous partager mon écran comme ça vous allez voir un PowerPoint pour le premier scénario.**

***Histoire #1***

***« Des parents hésitent à faire vacciner leurs enfants puissent qu’ils ont eu de fausses informations sur les vaccins. Ils se sentent submergés par toute l’information disponible en ligne. Ils ne savent pas où trouver les bonnes informations et quelle est la meilleure décision à prendre pour leurs enfants. À force de chercher en ligne sur des sites gouvernementaux et en comparant les informations avec les sites anti-vaccins, ils prennent la décision de faire vacciner leurs enfants».***

**Donc là, je vais vous présenter un vidéo, le vidéo est en anglais, mais il a des sous-titres en français. C’est un vidéo qui est disponible en ligne, qui circule déjà. Donc c’est vraiment un exemple qu’on veut vous montrer. Parfois il y a un petit délai entre le son et l’image. Hier quand on a fait le groupe de discussion, il n’y avait pas de problème, mais des fois il y en a un, donc j’espère que ce soir, ça va bien aller. Je vais commencer le vidéo.**

**Visionnement du vidéo #1** [Les deux parents hésitants- 6min43 à 10:03]

**Donc est-ce que vous avez pu comprendre le vidéo?**

002 et 003 : Oui.

**Donc est-ce que quelqu’un veut me parler en premier de ses premières impressions, du vidéo? Points forts, points faibles?**

004 : Ça ne me dérange pas de commencer. Les points faibles, je trouve que ça ressemble beaucoup au vieux documentaire qu’on voyait à l’école, un peu boboche, un peu comme mal fait honnêtement. Genre quasiment lire dans un script. C’est comme [INAUDIBLE 10:37] ce genre de trucs là. Je trouve qu’on voit vraiment que c’est dans une intention… Je ne dis pas que c’est *wrong*, mais c’est juste que je trouve c’est dur [INAUDIBLE 10:47] dans ce genre de vidéo-là. Je trouve que c’est mal fait, c’est vraiment dans une direction. Personnellement, sans avoir les contre-arguments, j’aime bien avoir des deux *sides,* un peu. Dans ce cas-là, je trouve que c’est très unidirectionnel. Donc ce n’est pas quelque chose qui m’épate.

**Pas assez nuancé, on va dire, un peu?**

004 : Ouais, c’est très scénario, genre au début on était comme ça, mais finalement on a changé d’idée. [INAUDIBLE 11:19] Je ne pense pas que ce soit assez convaincant(11:24).

002 : Si je peux enchaîner. Moi ce n’est pas nécessairement le genre de vidéo qui m’interpelle. C’est sûr que c’est très… bien c’est un peu typique comme [004] le mentionnait. Mais ce n’est pas non plus comme le genre de vidéo que je vais écouter, donc ça ne me rejoint pas.

**OK, parfait, quelqu’un d’autre? Est-ce qu’il y a quelqu’un qui a une opinion inverse ou la même opinion?**

003 : Bien disons que j’ai la même opinion. C’est vrai que ça fait vraiment comme les années 1990 quand je vois ça. Mais c’est sûr que je comprends qu’ils étudient les données, les risques. Mais ça ne change pas mon point de vue.

001 : C’est juste que le monde qui ont fait la publicité l’ont fait genre années 1980-1990, c’est une façon de faire. Typiquement[INAUDIBLE 12:49]. Pour le contenu, c’est vraiment prévisible genre dramatique, puis ça va vers tout est beau, la vie est belle, etc. Mais en plus je trouve que c’est un peu long comme publicité.

**Oui, donc trop long et trop prévisible, puis on voit juste un côté de la médaille, on va dire ça comme ça, si je comprends bien ce que vous dites? Puis vous ne vous reconnaissez pas non plus dans ce genre de scénario là?**

[Participants 001 et 002 font signe de ‘non’’ avec leur tête].

**J’avais une dernière question par rapport à celui-là, vous est-ce que vous regardez des vidéos en ligne? Puis si oui, c’est sur quelle plateforme que vous regardez des vidéos en ligne? Donc on pourrait commencer avec [002].**

002 : Bien en fait, moi c’est plus YouTube. C’est sûr qu’on ne parle pas nécessairement de vidéo informatif, sinon j’irais plus sur des sites gouvernementaux. Mais en général, c’est YouTube.

004 : Moi aussi Facebook, YouTube. C’est sûr que ça dépend de tel type de vidéo que je cherche. Clairement si ce sont des vidéos informatifs, YouTube va faire la source qui a fait le vidéo, ce n’est peut-être pas la meilleure source d’information tout le temps.

001 : Bien YouTube avant, mais maintenant, c’est rendu Facebook [INAUDIBLE14:35], ça dépend.

003 : Moi j’écoute souvent des documentaires dans YouTube. C’est sûr qu’il y a des documentaires de la France, puis des affaires que ça nous concerne moins, mais sinon pour des nouvelles d’ici c’est plus Facebook. Mais encore là, je fais attention parce qu’il peut quand même y avoir de fausses nouvelles.

**Super. Donc on va passer au vidéo #2. Je vais repartager mon écran.**

**Histoire #2**

***« Un médecin raconte qu’il a observé de ses propres yeux un enfant mourir d’une maladie qui est évitable par la vaccination. Il recommande donc aux parents de faire vacciner leurs enfants».***

**Donc ça aussi c’est un vidéo qui existe déjà en ligne qu’on a repris pour vous donner un exemple.**

**Visionnement du vidéo #2**  [témoignage médecin 16:01 à 17:32]

**Donc, premières impressions? Les points forts, les points faibles? Est-ce que quelqu’un veut commencer?**

002 : Je suis prête à commencer. Bien moi c’est sûr que ça, c’est le vidéo qui vient un petit peu plus me chercher quand ce sont des professionnels. Par contre, on s’entend que c’est sur un ton un peu monotone et plate, on ne se le cachera pas. Disons que je préfère Dr Quach quand elle fait ses présentations, mais moi c’est plus des professionnels qui viennent me chercher, puis justement qui racontent des faits vécus, bien moi ça m’interpelle un peu plus, de mon côté.

004 : C’est un peu comme [004] disait, le fait que c’est un professionnel de la santé, ça m’interpelle un petit peu plus, en fait beaucoup plus. Donc c’est un docteur, une expérience vécue, donc ça ne sort pas de nulle part. Puis aussi, c’est moins moralisateur un petit peu, ils vont moins dans la direction… bien la famille n’avait pas les informations nécessaires pour prendre une bonne décision, donc c’est moins moralisateur que l’autre côté. Puis aussi, c’est beaucoup plus court comme vidéo, tout à l’heure c’était… je ne me rappelle plus 3 ou 4 minutes, c’était beaucoup trop long, on perdait l’attention. Versus là, plus court, plus concis. C’est sûr que peut-être le ton serait un peu à revoir, mais sinon je pense que c’est beaucoup plus accrocheur et plus informatif.

**Merci, quelqu’un d’autre?**

003 : Bien moi le vidéo c’est sûr que son but ce n’était pas… ce n’est pas un film de Micheal Bay on s’entend, mais c’était clair, puis c’est selon son histoire, puis il ne veut pas nous l’imposer, il dit:« selon moi...», c’est vraiment ce qu’il a vécu, donc j’aime mieux ce vidéo-là. Puis c’est vrai que le ton est plate, mais bon, j’aime plus l’approche.

001 : Moi de mon côté, je trouve que c’est une vidéo un peu obsolète (19:53). Bah, si j’ai à mourir de la méningite, ça n’existe presque plus, même au tiers-monde. Et puis prouver qu’on excuse un manque d’information un moment où tout est branché, tout est sur internet, c’est quand même un argument qui n’est pas vraiment fort. Genre trouver qu’on excuse un manque d’information, ça, c’est obsolète comme information, de mon point de vue.

**OK, merci. Est-ce que pour vous ça vous rejoint davantage un médecin ou un autre professionnel de la santé par exemple : infirmière ou pharmacien? Est-ce que le type de professionnel qui raconterait un récit est important pour vous? Et si oui, juste me préciser lequel?**

002 : Personnellement je préfère que ce soit le médecin quoique pour moi un pharmacien aussi... pour moi ça a quand même de l’importance. Je ne dis pas qu’une infirmière, ça n’a pas d’importance, mais ça me rejoint plus des médecins parce que veut veut pas à mon avis ce sont eux qui ont travaillé beaucoup sur les recherches, mais encore là, je ne suis pas spécialiste. Mais sous toute réserve, moi ce sont vraiment les médecins.

004 : Moi aussi personnellement c’est [INAUDIBLE 21:25] du personnel de la santé. Les médecins me rejoignent un petit peu plus, mais quand même un médecin avec une attitude assez ouverte aussi. Par expérience, j’ai quand même été beaucoup dans les hôpitaux dans ma vie, les médecins sont très très fermés. Par exemple où est-ce que mon fils est né, on a eu une rencontre pour les vaccins justement dans la chambre médicale, puis justement Dre Quach... un de mes amis a eu une mauvaise expérience avec les vaccins RRO dans le fond, pour la rougeole. On venait un peu se rapprocher au Québec, donc les mauvaises expériences, puis j’ai expliqué à la madame que j’aimerais ça…dans le futur, c’est encore dans longtemps, mais je voudrais l’éloigner. Puis la madame était très fermée d’esprit : « non, non, non ne fait pas ça». Puis comme à la mode, elle est allée faire ses recherches, en allant voir la santé publique ontarienne, française et ainsi de suite. Elle a vu ce qu’il se faisait là-bas puis elle capotait un peu. Donc c’est pour ça que je dis, je pense que ça prend un professionnel de la santé ouvert d’esprit, dans ce sens qu’il soit capable de se faire *chalenger* parfois pour se faire demander des questions : « bien pourquoi c’est comme ça?».

001 : Bien un médecin ce serait comme n’importe qui parce que je pense qu’il faut un spécialiste [INAUDIBLE 22:51] peut-être même de type immunologiste. Et encore là, il faut quelqu’un qui a fait des recherches dans ce domaine-là. Parce que veut veut pas, moi ou n’importe quel généraliste, je crois qu’on est sur les mêmes niveaux de connaissances dans le domaine de tel ou tel virus. Bon, j’exagère un peu, mais moi ce que je verrais mieux, c’est quelqu’un qui a vraiment vécu la situation : une mère qui a vu son fils ou sa fille tomber malade, ou enfin, raconter une histoire fait vécu, c’est ça mon point de vue.

003 : Oui moi, c’est sûr que j’irais plus faire confiance à un médecin. C’est sûr qu’un pharmacien ne connaît pas le dossier médical de l’enfant, de la personne. Donc je pense que le médecin qu’on consulte depuis la naissance, c’est la meilleure personne qui peut répondre à cette question-là. C’est tout.

**Donc on a [001] qui a deviné la prochaine histoire qui est un parent qui raconte ce qui s’est passé avec son enfant. Donc la prochaine histoire est quand même… je veux vous avertir elle est quand même très touchante. Donc c’est une maman qui raconte qu’est-ce qui s’est passé, des complications d’une méningite avec son enfant qui est devenu aujourd’hui handicapé. Puis vous allez voir, ça touche beaucoup les émotions, il y a du piano derrière, c’est vraiment une ambiance, donc juste pour vous préparer psychologiquement au récit. Donc je vais vous partager mon écran.**

**Histoire #3**

***« Une mère témoigne des complications que son enfant a eues à cause d’une maladie évitable par la vaccination. Elle ne savait pas que le vaccin existait et parle de son expérience à l’hôpital et de l’impact sur sa vie actuelle avec un fils handicapé ».***

**Donc ça aussi c’est un vidéo qui circule en ce moment, celui-là est en frança**is.

**Visionnement du vidéo 3** (témoignage maman et enfant de 25:38 à 29:15)

**Vous avez bien compris l’histoire? Qu’est-ce que vous pensez à première vue de ce récit-là? Est-ce que ça vous interpelle? Les points forts, les points faibles?**

002 : C’est sûr que moi ça vient me chercher beaucoup. Oui je suis une personne émotive en général, mais parce que mon beau-frère il l’a attrapé la méningite quand il était plus jeune, puis il est rendu sourd et muet. Donc de voir que ça peut se rendre aussi loin, c’est sûr que ça m’interpelle. Puis avant d’être maman, je ne m’y connaissais pas trop dans les vaccins, donc la méningite et tout ça, je ne connais pas ça. Puis on me l’a offert pour ma fille plus jeune, mais il n’était pas obligatoire, il n’était pas dans les vaccins recommandés si on veut. Donc là, de me rendre compte que ça peut aller aussi loin, ça m’interpelle vraiment beaucoup.

**Si je me souviens bien, ce vaccin-là est disponible dans le calendrier de vaccination normal. Sinon, il y a une méningite que quand il y a une petite éclosion dans une ville, ils vont vacciner toute la ville d’un coup pour éviter que la méningite se propage. Mais au moment où son fils a attrapé la méningite, le vaccin n’existait pas, il n’était pas disponible à ce moment-là. Donc aujourd’hui c’est peut-être une histoire un peu plus désuète, on va dire. Mais c’est un peu l’impact quand même, de ne pas prendre un vaccin, ça pourrait avoir pour certaines maladies.**

004 : Oui bien moi ça me rejoint quand même beaucoup aussi. Je n’ai pas été malade à cause des vaccins, mais j’ai été malade quand j’étais kid. Donc de voir un petit enfant souffrir comme ça, c’est quand même très, très touchant. Je pense que comme n’importe quel traitement médical, les vaccins c’est de faire (31:18) un peu la balance des pour et des contre. Donc je trouve que c’est comme un gros argument dans la balance pour faire vacciner ses enfants, selon moi. C’est touchant.

001 : Même moi quand elle a dit que le vaccin n’existait pas, j’ai trouvé ça un peu bizarre parce que le jeune a l’air d’avoir 12-13 ans à peu près, donc comme je disais moi personnellement un de mes oncles est mort de la méningite, ça fait longtemps, et puis même dans les pays du tiers-monde le vaccin existe pour la méningite. Je ne savais pas que c’était au choix, par contre.

003 : C’est un bon petit vidéo quand même triste, c’est sûr que c’est vraiment triste comme histoire. Il faut regarder les deux côtés, puis je pense que le côté est quand même assez pesant pour dire : «oui, il y a un gros effet là-dessus». Donc c’est sûr que moi je n’hésiterais pas à faire vacciner mon enfant. D’ailleurs il est vacciné mon fils. Puis là-dessus je suis d’accord, il faut y aller avec la logique. Aussitôt qu’il le pogne...bien ce n’est pas aussitôt qu’il le pogne, il ne faut pas virer fou, mais il a de fortes chances… bien pas de forte chance, mais ça peut virer quand même assez drastique.

001 : J’ai oublié, j’ai parlé de la méningite, je n’ai pas parlé de la vidéo. Pour la vidéo je trouve qu’elle est longue, donc peut-être utilisé… couper un peu parce qu’elle est un peu longue.

**Est-ce que vous pensez qu’on devrait changer de maladie, par exemple, on pourrait parler des impacts de la rougeole de cette façon-là? Ou, des impacts d’autres maladies évitables par la vaccination? Tétanos, c’est plus rare que les gens attrapent le tétanos, mais une autre maladie évitable par la vaccination, est-ce que ça vous interpellerait plus? Ou, la méningite, ça vous interpelle quand même?**

002 : Bien moi, c’est sûr que je les trouve toutes aussi pertinentes(33:55). C’est sûr que peut-être moins le tétanos, mais on s’entend, je les trouve toutes aussi pertinentes, mais je t’avoue que celui de la méningite vient plus me chercher parce que si ça n’aurait pas été de mon beau-frère, peut-être que j’aurais été hésitante à me dire : « ouais, mais y a-t-il vraiment des chances que ma fille attrape ça?». Je pourrais dire : « on ne l’attrape pratiquement jamais, il n’y a pas grand monde qui l’attrape». Mais là, sachant que j’en ai eu dans la famille de mon conjoint, bien on dirait que ça m’a plus interpellé. Mais là en voyant le vidéo… Même si mon beau-frère ne l’avait pas eu, je pense que je l’aurais fait vacciner.

**Quelqu’un d’autre?**

001 : Moi je suis vacciné contre le tétanos, ce sont des vaccins de 10 ans. Donc c’est sûr qu’il y a certaines maladies qui n’existent plus comme la tuberculose, mais il y en a d’autres qui reviennent comme la typhoïde et puis la rougeole et si mes connaissances sont bonnes elle se transforme après en zona. Je pense même que le gouvernement a commencé à vacciner contre le zona pour les gens d’un certain âge. Donc c’est sûr qu’il y en a certaines qui vont revenir, il y a certaines qui vont exister. Puis il y en a qui comme la tuberculose, je ne vois pas l’utilité de vacciner contre ça, parce que ça a complètement disparu.

**Donc, parler d’un vaccin où il y a des éclosions en ce moment, ce serait plus pertinent, c’est ce que je comprends?**

[ le participant 001 fait signe ‘’oui’’, de la tête].

**Quelqu’un d’autre? Donc tout le monde est du même avis, c’est ce que je comprends?**

[Participant 003 fait le signe du pouce en l’air]

**Maintenant on va parler des récits en général, quel récit vous a le plus interpelé et pourquoi? Donc on va commencer avec 002.**

002 : Bien c’est sûr que clairement, c’est le dernier, quand on vient toucher les émotions puis encore plus quand tu as un enfant, on dirait que ça vient vraiment chercher au fond du cœur. Donc c’est clairement le dernier.. Puis comme je le disais c’est surtout parce que c’est… bien pas surtout, mais admettons que quand tu as un enfant, ça nous interpelle plus en tant que parent, c’est sûr. Puis il faut vacciner dès le jeune âge. Donc c’est vraiment la dernière, sans aucun doute.

004 : Moi c’est un mélange de la dernière puis celle du docteur [36:41]. Je suis quand même quelqu’un de très rationnel dans la vie, donc je dirais un mélange des deux. C’est sûr que depuis que j’ai un enfant, le côté émotionnel des fois il prend un peu le dessus.

001 : C’est sûr que quand on parle du concept, les 3 sont touchants, mais quand on parle de la façon, pour la première ça fait vraiment une histoire hollywoodienne où il y a des fleurs derrière, un background, etc. La deuxième ça fait un médecin avec une blouse blanche qui nous raconte qu’on doit se brosser les dents. La troisième par contre, pour moi, c’est la meilleure, mais il faudrait peut-être la retravailler. Utiliser les moyens de connaissance des spécialistes en audiovisuelle pour la rendre encore plus brève (37:92).

**Puis un peu plus courte de ce que je comprends aussi parce que tantôt vous avez dit qu’elle était longue.**

001 : Exact parce que les publicités longues, ça nous pousse à zapper.

003 : Bien moi, c’est le troisième que je trouve le plus intéressant. Les deux autres, on dirait que c’est plus *stagé*, mais le troisième, *stagé* ou pas, c’est la réalité aussi. J’aime mieux la troisième.

**Puis le moins intéressant, pour vous c’était le premier pour tout le monde, de ce que je comprends, ça ne vous interpelait pas du tout.**

[ Les participants 002, 003, 004 font signe ‘’oui’’ de la tête].

**Est-ce que vous avez des suggestions pour rejoindre des parents… comme retravailler des vidéos ou des informations qu’on devrait inclure dans un vidéo pour mieux outiller les parents à prendre une décision? Peut-être une décision avec des informations nuancées, auriez-vous un exemple qui irait dans ce sens-là? Comme un vidéo parfait pour vous, avec le type d’information. On pourrait commencer avec [002].**

002 : C’est rare que j’aie moins de rapidité à répondre. Écoute je ne le sais pas trop, honnêtement, vite comme ça. Je ne sais pas si je peux laisser ma parole.

**Oui, pas de problème. Est-ce que quelqu’un est prêt à répondre à une idée?**

004 : Je peux y aller. Je pensais à un vidéo short qui explique quand même assez… par un professionnel de la santé, les bienfaits de la vaccination. Mais qui donne des liens aussi vers des capsules extérieures admettons pour les différents types de vaccins, puis si la personne est intéressée, elle peut aller voir en détail les pour et les contre. Un peu comme un médicament, de donner les effets secondaires du vaccin, puis les complications possibles. Mais aussi expliquer c’est quoi [INAUDIBLE 39:48] les complications. Mais aussi d’expliquer qu’est-ce que le vaccin prévient, puis donner son utilité (39:53). Donc ça je pense que ça permettrait aux gens de voir qu’il y a quand même des contre, mais les pour sont... [INAUDIBLE 40:03].

001:Oui comme je le disais toutes les histoires où il y a une perte humaine ce sont des histoires qui touchent ou bien de la souffrance, ça va avec. Mais pour ce qui est de l’information qui est véhiculée dans le fond quand quelqu’un qui parle comme ça, on a tendance à s’enfuir surtout quand on n’utilise pas une voix professionnelle. On n’a pas tous une belle voix, c’est malheureux, mais des fois, même si la personne souffre vraiment, mais le fait de l’entendre parler, on n’aime pas sa voix ou bien elle ce qu’elle est en train de dire peut-être que ce n’est pas pertinent. Donc moi, je serais plus pour une voix professionnelle avec des personnes (41:09), on utilise le même procédé visuel, mais peut-être sans la voix ou sur la musique, d’autre chose professionnelle avec des personnes.

003 : Moi la vidéo, c’est sûr que ce serait mieux qu’il soit plus court. Puis le fait aussi de dire que le message vient du Collège des médecins, je trouve que ça donne de la crédibilité. Un message qui provient du gouvernement du Québec, le monde sont plus hésitant là-dessus. Mais quand ça vient du Collège des médecins, puis que tu vois que c’est prouvé. Puis une vidéo aussi pas trop émotive par ce que ça donne… peut-être que créer de la peur pour rien aussi. Oui, c’est épeurant, mais pas le temps de stresser puis go il faut faire…

**OK, donc un peu plus atténué, on pourrait enlever le piano.**

003 : Oui parce que j’ai quasiment pleuré. C’est jouer avec les émotions.

**Oui, oui, oui, bien moi aussi la première fois que j’ai vu le vidéo, j’avais les larmes aux yeux. Mais non c’est vrai qu’il y a beaucoup d’intensité au vidéo. Donc pour vous ce serait de l’atténuer un peu, puis de rajouter des informations avec par exemple le Collège des Médecins qui appuie la vidéo, et tout.**

003 : C’est ça.

**Pour [002]?**

002 : C’est sûr que vous m’avez tous inspiré. C’est sûr que comme tout le monde dit, moi aussi je la mettrais plus courte, je suis totalement d’accord. Puis bien moi je un peu sur le fait que c’est bon qu’il y ait de l’émotif parce que veut veut pas… bien en tout cas moi je suis beaucoup sur Opération Enfant-Soleil, puis veut veut pas, les gens appellent et donnent par rapport à ce qu’ils voient à la télé, puis c’est souvent très émotif, le téléthon, on ne se le cachera pas. Donc c’est sûr que c’est un élément qui fonctionne bien, mais je pense que ce qui pourrait être bien à la fin, je suis d’accord pour le Collège des Médecins qu’il mentionne ça, mais à la fin il pourrait au pire donner une statistique, il y tant de personnes… les pourcentages que les gens attrapent cette maladie-là. Mais à la fin, peut-être préciser : « on ne veut pas que ça soit le vôtre» ou quelque chose comme ça, en voulant dire : «oui c’est très nuancé, très émotif, mais c’est juste pour prévenir que ça arriverait, puis que ça donne sur notre enfant bien…». Moi, c’est comme ça que je le vois, puis que j’aimerais le voir en fait, mais c’est ma perception.

001 : Excusez, c’est juste que quand j’ai entendu ça, moi personnellement, l’histoire de sensibiliser ou de transmettre l’information, ça ne marche pas vraiment. Il faut vraiment être… Je dirais du genre:« vous allez crever», il faut faire peur, c’est la peur qui pousse le monde. Si par exemple vous dites : «OK si vous continuez à fumer, vous n’allez pas pouvoir monter telle montagne, vous allez être essoufflé», non «vous allez monter directement, vous allez dans un cercueil», ça c’est plus efficace, à mon point de vue. Pas parce que ce n’est pas correct, ça va passer dans le carré rouge ou quoi que ce soit, mais c’est plus efficace.

**Merci. Est-ce que pour vous ça a vraiment de l’importance que la personne ait réellement vécu la situation ou si on prenait un acteur par exemple, ça serait quelque chose qui serait correct? Est-ce que vous aimez mieux une vraie personne qui témoigne ou un acteur, c’est plus ça la question?**

002 : Pas d’acteur. Pas d’acteur. Pas pour moi.

**Est-ce que vous êtes tous du même avis?**

003 : Bien c’est sûr qu’il n’y a rien qui peut remplacer la vraie personne. C’est vraiment ça qu’elle a vécu, l’acteur ne peut pas vraiment remplacer… Il ne peut pas vraiment acter ça, s’il n’a jamais vécu ça.

004 : Moi je suis un peu partagé, un peu comme [001] disait. S’il n’a pas une belle voix ou s’il ne passe pas bien à l’écran, je pense que même si tu as vécu l’histoire, tu peux peut-être avoir de la difficulté à le retranscrire dans une vidéo. Je suis comme partagé entre les deux honnêtement, donc ça prend comme quelqu’un qui a vécu l’histoire, mais qui est quand même bon pour le transmettre aux autres. Sinon peut-être qu’un acteur ou [INAUDIBLE 45:50] pour transmettre l’information.

001 : Un acteur, c’est sûr que ça prend quelqu’un qui a vécu ça ou peut-être une célébrité, ça dépend le budget que vous avez, mais quelqu’un qui sort de l’institut du cinéma ou quelque chose, non, ça ne fait pas sérieux.

**Sur quelles plateformes vous aimeriez voir ce type de vidéo? Est-ce que Facebook, YouTube, un site du gouvernement, où est-ce que vous aimeriez voir ce type de vidéo?**

002 : Bien moi il faudrait que ça soit… c’est triste à dire, mais des publicités que je n’ai pas trop le choix de regarder pour pouvoir regarder ce que je veux. Parce que veut veut si je le vois sur Facebook, je ne serai pas du genre à arrêter pour ce genre de vidéo-là. J’essaie de garder mon train-train agréable sans… Je ne le sais pas, des fois Facebook, il y a tellement d’affaires négatives que je ne regarde pas ce genre de vidéo là.

**Parfait, quelqu’un d’autre?**

003 : Bien c’est sûr que ce serait mieux sur Facebook, tu ne vas jamais taper ça sur YouTube : «vaccination...», mais si c’est une annonce, c’est comme une manière de nous forcer à regarder, bien c’est sûr que ça serait plus là, sous forme d’annonce, ça peut être sur YouTube, mais pas juste la vidéo toute seule, de même.

**Quelqu’un d’autre?**

001 : C’est sûr qu’il faut la mettre là où il y a du monde : Facebook, YouTube ou même un site porno, je ne le sais pas, là où il y a du monde, donc on ne va pas la mettre dans un site de gouvernement, personne n’y va. Peut-être pour regarder ses impôts, mais c’est sûr qu’il faut y aller, là où il y a du monde.

**[004], est-ce que c’est la même chose?**

004 : Oui, bien oui… j’avoue que peut-être Facebook a plus de chance par exemple, ça peut rejoindre du monde, sur le site du gouvernement je ne pense pas que le trafic est si énorme que ça. Même si sur Facebook, ça peut être relié à une page du gouvernement parce que ça vient de [INAUDIBLE48:37]. Mais clairement qu’il y a beaucoup de monde comme [002] vient de le dire qui vont faire skip [INAUDIBLE 48:46]

**Donc spontanément vous n’allez pas faire une recherche YouTube pour trouver ce genre de vidéo là, puis il faudrait vraiment qu’il apparaisse de façon forcée dans votre fil d’actualité Facebook, par exemple, ou d’autres sites.**

002 : J’ai pensé à une petite chose, avant de tomber enceinte, j’ai été aussi à l’hôpital, ils donnent des informations, c’était mon premier bébé. Donc ça ne pourrait pas être mauvais qu’on nous montre des vidéos parce qu’ils nous disent tellement d’affaires par rapport à l’accouchement, puis à quel point ça peut être intense, mais de prévenir justement, puis de dire : « bien écoutez pour les vaccins, voici ce qui pourrait arriver». Moi personnellement, en plus enceinte, donc je serais encore plus interpellée. Je le suis déjà, mais on dirait que ça serait comme fois 1000 à cause des hormones(rire).

**Donc comme par exemple, à un rendez-vous médical avant d’accoucher, on vous montre le vidéo dans le cabinet du médecin.**

002 : Non, peut-être pas là. Non, mais, il y a des sessions qu’on peut aller, puis qui donnent de l’information par rapport à l’accouchement. Ce n’est pas avant un rendez-vous. C’est vraiment une session d’information, je comprends que ce n’est pas tout le monde qui y va, mais moi je trouvais ça important, puis on était quand même une dizaine de parents, puis c’était comme ça régulièrement qu’ils faisaient. Donc je pense que ça peut interpeler beaucoup de monde.

**Est-ce que c’était comme des cours prénataux?**

002 : Non, même pas. Ça, ça pourrait être une option aussi. Mais non, ce n’était pas des cours prénataux, c’était vraiment une session informative à l’hôpital : quoi apporter pour votre accouchement, comment ça peut se dérouler, c’était vraiment une session informative.

**Je comprends. Est-ce qu’il y avait d’autres choses par rapport aux vidéos ou la plateforme pour recevoir le vidéo, avez-vous d’autres idées?**

003 : Bien c’est sûr que d’avoir toujours la même annonce qui réapparaît à un moment donné, ça va écœurer le monde. Donc juste doser le nombre de fois qu’il devrait apparaître.

**Donc pas trop payer cher pour que ça arrive dans votre fil d’actualité, tous les jours.**

003 : Parce que selon moi, quand on force quelqu’un, il va faire l’inverse, ça, c’est selon moi.

**Est-ce que vous aviez d’autres choses que vous aimeriez partager aujourd’hui avec moi par rapport aux vidéos qu’on vous a montrés? J’ai peut-être une dernière question avant, est-ce que format vidéo, c’est quelque chose qui vous plaît ou est-ce que par exemple si on faisait un petit script qu’on mettrait sur les médias sociaux écrit, ça vous rejoindrait plus? Ou est-ce qu’une bande dessinée, par exemple ça vous rejoindrait plus? Ou, une image quelconque, une image avec plusieurs informations? Est-ce que le vidéo est le moyen qu’on peut le plus vous atteindre ou atteindre la population avec ou il y a d’autres médiums qui seraient possibles selon vous?**

002 : Moi c’est vraiment les vidéos, le texte, je sais que je ne prendrai pas le temps de le lire. Les BD et tout ça, je skip assez rapidement, donc ça ne me rejoint pas pour moi, ce serait vraiment les vidéos.

004 : Pareillement aussi, à moins d’être vraiment à la recherche d’une information précise, ça ne me dérange pas d’aller lire, mais si c’est quelque chose qui m’est balancé, je pense que le vidéo serait plus rapide, plus d’information dedans.

003 : Sous forme de vidéo, mais aussi accompagné d’un tableau pour donner des faits, de vraies choses. Quand je vois un tableau, pour moi c’est quelque chose de sérieux. La vidéo aussi, pour accrocher l’œil, puis ensuite le tableau.

001 : C’est sûr que le texte, je ne pense pas qu’une personne va le lire. Bien pour le contenant qu’il soit en image, en vidéo ce n’est pas ce qui est le plus important, c’est plus le fond parce que par exemple si vous payez des publicités sur YouTube, il faut mettre que vous avez 5 secondes pour faire passer un message, sinon le monde va cliquer sur suivant. Et c’est la réalité, la plupart du monde clique sur suivant, donc il faut que le fond se passe dans les premières 5 secondes. Maintenant vidéo ou… Bien c’est sûr que moi personnellement, c’est plus du vrai monde, mais si on ne peut pas, on peut mettre des bonhommes comme ça.

**Donc moi ça fait le tour des questions que j’avais pour vous ce soir. Est-ce qu’il y a d’autres choses qu’on n’a pas parlé que vous aimeriez discuter ou vous avez des questions et interrogations?**

001 : La sorte de musique qu’il y a en background.

**OK, l sorte de musique. Quel genre de musique ça serait le mieux? De la musique avec des effets sonores avec une intensité ou de la musique douce, quel genre de musique?**

003 : Certainement pas comme dans drôle de vidéo. Bien ça dépend du genre de vidéo, si c’était comme la vidéo de tantôt avec le petit gars Benjamin, bien la musique était correcte, ce n’est pas le temps de mettre du classique aussi, ce n’est pas le temps de mettre du rock, mais pas trop fort, assez bas pour qu’on entende les gens parler.

001 : Moi, c’est plus pour du classique dramatique. Il y a eu à un moment donné une série de publicité de la SAAQ pour discuter de la conduite en état d’ébriété, et puis il avait utilisé, je pense, un morceau de [INAUDIBLE55:57] et c’était vraiment fort comme publicité, la musique en arrière plan était vraiment… Et c’était un truc classique.

**Est-ce que quelqu’un d’autre a un avis sur la musique?**

[Les participants sont silencieux]

**Donc avez-vous d’autres commentaires, questions par rapport à ce qu’on a discuté ce soir?**

003 : Non, pas moi.

**Focus group 3**

**À quel point vous utilisez les médias sociaux : internet, Facebook, Twitter et tous les nouveaux réseaux sociaux que je connais moins. Mes deux enfants ont maintenant 11 et 13 ans, donc je suis dans un autre âge. Ça fait longtemps que les décisions de vaccination ont été prises. Mais donc les réseaux sociaux que vous utilisez pour vous renseigner. Donc [001] je vois aussi que tu as un ou une spectateur additionnel, il a l’air bien intéressé.**

001 : Et puis réseaux sociaux, j’utilise pas mal juste Facebook, je ne suis pas très réseaux sociaux beaucoup, puis Pinterest, mais ce n’est pas vraiment… Ce n’est pas vraiment comme Instagram ou quoi que ce soit. Donc je ne suis pas beaucoup sur les réseaux sociaux, sauf Facebook.

002 : Moi c’est [002]. Puis ce qui est des réseaux sociaux, moi aussi je ne suis pas très réseaux sociaux, j’utilise beaucoup Facebook, Messenger, aussi beaucoup Whatsapp, Instagram un peu, mais je ne publie pas moi-même, mais j’ai un compte Instagram, puis c’est pas mal ça. Twitter, non, pas du tout.

**L’utilisation des réseaux sociaux.**

003 : Non, les réseaux sociaux, c’est seulement pour contacter mes amis qui ne sont pas proches de chez-moi, seulement pour faire des groupes. Mais aucun pour avoir de l’information ou quoi que ce soit, parce qu’il y a trop de cochonneries.

**L’objectif du projet, vous n’êtes peut-être pas représentatifs, mais on sait que les parents en général prennent beaucoup d’informations sur les réseaux sociaux, puis qu’il y a beaucoup d’informations sur les vaccins qui circulent. Donc nous on a développé des interventions basées sur des témoignages ou des histoires. Donc c’est sur quoi on aimerait avoir votre avis, puis vraiment on veut votre avis sincère. Donc si vous trouvez que ce n’est pas bon que vous ayez des critiques, il ne faut pas du tout se gêner pour nous en faire part. C’est dans l’optique de l’améliorer donc on a plusieurs groupes de discussion prévus, on va essayer d’améliorer notre intervention avec les propos des parents ciblés. On va présenter un PowerPoint, mais ça ne sera pas tout le long, juste pour vous faire la présentation de la première histoire.**

**[003] est-ce que tu avais une question avant qu’on débute?**

003 : Oui, ça se peut qu’à 15h30 j’aie à partir pour 5 minutes, quelque chose du genre.

**Parfait, bien noté.**

**Histoire #1**

« *Une mère hésite à faire vacciner son enfant et fait des recherches en ligne sur le sujet* (-mais elle a beaucoup de la misère à trouver de la bonne information parce qu’il y a beaucoup, beaucoup d’information sur internet. Cœur de l’histoire : sa cousine est infirmière, donc elle va demander de l’information à sa cousine). *Sa cousine infirmière l’informe sur le sujet et répond à ses inquiétudes*. (elle obtient des réponses à ses inquiétudes sur la vaccination. Puis après que sa cousine ait répondu à ses questionnements, elle est maintenant…elle se sent plus apte à prendre une décision éclairée sur la vaccination de son enfant). *La mère, maintenant mieux informée sur le sujet peut prendre des décisions plus éclairées pour la sécurité et la santé de son enfant*».

**Donc on aurait un petit vidéo à vous montrer qui est un peu dans le même style que cette histoire-là, mais pas tout à fait pareil. Parfois il y a un petit délai entre l’image et le son avec la plateforme Teams selon combien il y a d’individus sur la bande passante de l’institut avec nous en même temps. Donc on va espérer qu’il n’y ait pas trop de délais.**

**Visionnement du vidéo #1** [ Les deux parents hésitants 7:37 à 10:57]

**En fait on vous a présenté le scénario, puis le vidéo, on s’excuse, il est en anglais, parce qu’on n’a pas trouvé un comparable en français. Mais l’idée qu’on voulait, c’était vraiment d’illustrer des parents qui ont fait face à de la désinformation en ligne, puis qui après ont trouvé d’autres informations, puis ils ont changé leur opinion.**

**Donc là d’abord, j’ai une première question très générale, c’est quoi vos impressions de cette histoire-là ou de ce vidéo-là? Est-ce que vous vous reconnaissez dans ces parents-là?**

001 : Personnellement, non. Je n’ai jamais eu de… C’est sûr qu’il y a toujours des gens qui vont mettre des faits négatifs, ceux qui ont peur de ça, mais je n’écoute pas vraiment ce que les autres vont dire, ou je ne vais pas aller rechercher sur internet, parce que justement il y a souvent des informations qui sont fausses. Puis on dirait que plus que tu lis, plus que c’est dramatique. Un peu comme quand on cherche, on a un bobo quelque part, puis on finit avec un cancer. Donc je ne me fie à ces informations-là en fait.

003: Moi c’est un peu pareil aussi. Mais par contre, si c’est une information dont j’ai vraiment besoin, je ne me fierais pas juste à un site. Surtout pas à un groupe qui est anti-vaccin déjà à la base. Je vais y aller sur 4-5-6 sites différents. Je vais aller sur des sites du gouvernement surtout, par des organismes de la santé pour être sûr de c’est quoi l’info. C’est sûr que s’il est allé sur un site anti-vax, bien c’est sûr qu’il est anti-vax, il a eu l’info qu’il voulait. Parce que sur internet, tu veux trouver de l’info que quelqu’un a mangée, une vache crut, tu vas le trouver. Donc, c’est ça.

002 : C’est ça il y a des sites tout dépendant de la réponse que tu veux, tu vas trouver la réponse peu importe. Ce qui est important, c’est de savoir justement la crédibilité des sites internet d’où on va chercher l’information. Donc moi non plus personnellement, je ne me fie pas beaucoup aux réseaux sociaux. Par contre, eux dans le vidéo, je pense qu’ils mentionnaient aussi le fait qu’ils avaient été peut-être… ils avaient entendu à travers des amis. Donc si par exemple amis, famille, là ils commencent peut-être à parler de ça… Je sais qu’il y avait par exemple une vague qui disait que la vaccination pouvait causer l’autisme. Donc ça c’est quelque chose qui est assez répandu, pas juste sur les réseaux sociaux, j’ai des amis aussi qui le pensent, donc ils ont décidé de ne pas vacciner leurs enfants. Donc ça pèserait peut-être un peu plus sur la balance pour comme créer un doute. Ça serait plus les amis que les réseaux sociaux.

**Donc je comprends bien l’angle trop internet, «je cherche de l’info en ligne», c’est peut-être moins pertinent ou moins... On devrait peut-être plus centrer l’histoire sur un parent qui parle avec d’autres parents de son entourage, puis que là il entend une histoire d’horreur par un contact. Puis là, ça l’inquiète que le focus sur « je cherche sur Google et je tombe sur un peu n’importe quoi».**

003 : Le problème c’est que la personne de ta famille avec qui tu parles, elle, elle est probablement allée chercher son info sur un site anti-vax. Mais là, le réflexe qu’on devrait avoir c’est : «OK, tu sors d’où?». Mais là, bien ça va commencer à faire des chicanes : «tu ne me crois pas». En tout cas bref…

001 : Mais je pense que votre histoire par rapport à la recherche n’est pas nécessairement fausse parce qu’on le fait tous un peu quand même. Dans cette option-là, eux ils ont juste regardé internet, on le fait tous un peu, mais après ça on va quand même rechercher l’information à la bonne place aussi. Il y en a qui font que ça, puis c’est dur comme faire, ils l’ont lu quelque part, puis c’est la réponse.

003 : Oui, bien c’est ça c’est parce qu’ils cherchent cette réponse-là.

001 : C’est ça.

002 : C’est ça le problème.

003 : Ils ont leur idée, mais ils veulent juste se faire autoconvaincre, dans le fond

002 : Parce que si tu cherches ‘’vaccin danger’’, ‘’ vaccin effets secondaires’’, c’est sûr que tu vas trouver une panoplie d’information. Mais c’est ça, c’est de voir où sont les sites crédibles?

**Parfait. Puis c’est sûr que cette histoire-là date un peu, je pense que le tournage a eu lieu, il y a une dizaine d’années. Donc là on sait qu’il y a quand même… Les gens sont peut-être plus au courant des fakenews, puis toute la désinformation qu’il y a en ligne, je pense que c’est plus présent à l’esprit. Donc je comprends que vous ne vous reconnaissez pas nécessairement non plus dans ces personnes-là?**

[Les participants 001 et 003 font ‘’non’’ de la tête].

**L’approche naturelle par rapport à la santé ou l’effet de groupe, est-ce que c’est quelque chose que vous avez connaissance, des gens autour de vous qui…**

003 : Moi l’effet de groupe, ça ne m’affecte pas bien, bien (rire). Non, je vais être dans un groupe, tout le monde va croire à quelque chose, je vais aller double vérifier comme je le disais avec plein d’info, puis surtout des sites reconnus. Avec de l’information reconnue.

**Puis est-ce que le fait de faire une histoire où on présente des sites reconnus… Parce que quand on travaille avec des personnes ou des parents qui hésitent par rapport aux vaccins, des fois on se fait dire : «bien les sites du gouvernement, c’est trop vendu. Ce n’est pas assez nuancé, ça présente l’information trop simplement», donc on va contraster ou comparer… Les gens c’est comme s’ils se disent : «bien là il y a le provaccin du gouvernement, puis là je vais aller lire sur des sites anti-vaccins pour comme avoir l’autre côté de la médaille», puis là on n’est pas dans le même niveau d’information, puis ça peut être inquiétant.**

001 : Oui, je comprends. C’est sûr que cette histoire-là serait plus à jour, mais ça ne me représente pas moi. Mais il y en a que justement ils vont aller voir les deux extrêmes, puis c’est sûr que comme site internet… C’est sûr que si on regarde juste le gouvernement, c’est sûr qu’ils sont juste provaccin. Donc il n’ont pas les deux mesures, admettons.

003 : Le pire c’est que dernièrement, je suis allé chercher justement pour des infos sur des effets secondaires d’un vaccin. Quasiment toute la semaine, j’ai regardé là-dessus. Je n’en ai pas trouvé tant que ça. Puis je ne suis pas arrivé sur un site d’anti-vax. Puis je cherchais exactement les effets secondaires pour les vaccins de la diphtérie, rougeole et tous ceux qu’ils donnent à nos enfants quand ils sont jeunes. Puis j’avais entendu que la fièvre jaune pouvait donner la jaunisse, puis des problèmes de foie, même, que j’ai trouvé quelqu’un qui est mort de ça. Mais la fièvre jaune, ce n’est pas un vaccin qu’on donne tout le temps, c’est juste quand tu vas au Pérou ou quelque chose du genre. J’ai vraiment cherché et je n’ai pas trouvé d’infos négatives tant que ça. Donc ce n’est quand même pas si pire.

**Bien je pense qu’entre autres depuis la COVID, il y a quand même un effort des plateformes pour essayer de contrôler ces discours-là, donc ça se peut que l’information… Comme entre autres Facebook a fermé plusieurs pages, à cause qu’il y avait du contenu contre les vaccins. Donc ça devient des groupes fermés qu’on ne peut pas nécessairement tomber dessus directement.**

**Je propose qu’on passe à l’histoire #2 tout de suite.**

**Histoire #2**

*«Le succès des vaccins amène la population à ne plus voir les dangers liés à la maladie, mais plutôt ceux liés aux vaccins. Un médecin raconte qu’il a observé de ses propres yeux des enfants mourir de maladies qui sont désormais évitables par la vaccination. Le médecin recommande à tous de s’informer au sujet des vaccins et de vacciner leurs enfants avant qu’il ne soit trop tard».*

**Donc là, on a un vidéo en français cette fois-ci.**

**Visionnement du vidéo#2** (témoignage du médecin de 20:21 à 21:34)

**Ça a lagué beaucoup, j’espère que vous avez pu le voir sans trop de problèmes. Tant mieux si c’était juste de mon côté.**

003 : On peut fermer nos caméras pendant qu’on regarde le vidéo, comme ça, ça aide beaucoup.

**Oui, c’est vrai.**

003 : C’est pour ça que je l’ai fait.

**Ma première question pour vous c’est qu’est-ce que vous pensez du fait que c’est un professionnel de la santé qui partage son histoire?**

002 : Je trouve que c’est plus crédible. Je vais plus être portée à écouter, à prendre son opinion en considération. Je trouve que ça joue un rôle positif.

003 : Oui, moi aussi.

001 : Même chose pour moi. C’est sûr qu’un médecin est pas mal plus qualifié que souvent un site internet avec des opinions ou quoi que ce soit. Donc c’est sûr que je vais me fier pas mal à l’avis d’un médecin qui a justement fait des études là-dedans. Puis l’expérience personnelle de l’avoir vu lui-même, aussi. De pouvoir témoigner en faveur du vaccin.

**Est-ce que vous auriez eu la même réaction si ça avait été un autre type de professionnel de la santé comme une infirmière, par exemple? Ou, vraiment le médecin a un…**

003 : Infirmière, même chose pour moi.

002 : Oui, moi aussi.

001 : Oui, parce qu’elles en voient tellement elles autres avec..

003 : Elles sont comme sur le front, les infirmières…

001 : Pas mal, oui.

**Puis un pharmacien?**

001 : Oui, parce qu’ils ont quand même une formation sur les médicaments. Donc ils ne sont pas non plus… c’est sûr que s’il contredit le médecin, je ne vais peut-être pas faire la même chose. Mais s’ils corroborent avec les mêmes informations, oui. Un pharmacien est quand même qualifié.

003 : Oui, souvent je pose énormément de questions au pharmacien, puis il y a beaucoup de réponses qui arrivent.

**C’est sûr qu’effectivement, ils connaissent beaucoup en médicament, puis souvent plus que les professionnels de la santé, parce qu’ils se centrent là-dessus, mais il y a quand même des fois un biais parce qu’ils en vendent. Il y a profit financier, donc c’est parfois plus délicat. Donc le fait que ça soit un professionnel de la santé ça vous interpelle comme parent, davantage que si c’est un autre parent?**

001, 002, 003 : Oui.

**Je comprends que vous avez confiance plus dans l’information transmise par un professionnel de la santé que par un parent?**

001 : Oui.

**Puis comme question peut-être un peu sensible, dans le contexte de la COVID, on a vu quand même certains professionnels de la santé exprimer des opinions ou avoir une position qui est très divergente de ce qu’on entend habituellement, est-ce que ça vous interpelle davantage ou ça vous inquiète davantage?**

001 : C’est sûr que pour la question du COVID c’est complexe, parce que c’est un vaccin qui est sorti quand même… Normalement ils disent que c’est à peu près un 5 ans d’étude, mais là c’est sorti vite. Je vous dirais que je l’ai fait quand même parce que c’est ça qu’ils suggèrent pareil de faire. C’est sûr que s’ils demandent de vacciner les bébés, bien je le ferais, je l’ai faite enceinte en fait, une dose. Mais ça reste que j’ai une crainte intérieure des études qui vont suivre plus tard admettons. J’espère avoir fait le bon choix, mais j’ai eu une petite peur quand même.

002 : Pour moi la COVID, c’est vraiment différent dans mon opinion qui est différente des autres vaccins, pour les autres maladies. Je ne sais pas si vous voulez avoir mon opinion juste pour la COVID? Je ne sais pas en fait si je vais vacciner ma fille. Parce que justement le vaccin est trop nouveau, il y a trop d’enjeux… Des enjeux économiques aussi, comme la course contre le vaccin. Puis tout le monde pousse vraiment pour leur vaccin sans vraiment… On entend beaucoup sur le positif, mais… Je suis encore incertaine. Par contre pour les autres maladies, pour tous les vaccins, ça fait longtemps qu’ils sont administrés, donc j’ai beaucoup plus confiance, pour ma fille. Je me suis fait vacciner moi pour la COVID, les deux doses, mais pour ce qui est de ma fille, je ne suis pas encore certaine que je le ferais.

003 : Je suis entièrement d’accord avec [002] là-dessus. Les autres vaccins, on les vaccine ça fait quoi? Quasiment 70 ans. Ce sont des vaccins qui ont été quand même prouvés, je les ai eus, j’ai 46 ans. Il n’y a pas eu d’effets secondaires. Ça, ce n’est pas un vaccin, c’est un ARN-messager, c’est complètement différent. Ça modifie l’ADN. Je ne veux pas rentrer quelque chose dans mes filles qui n’ont pas été ‘’éprouvé’’ pendant une dizaine d’années. Puis qu’on ne sait pas, si ça va faire une rétroaction à long terme. Ça n’a aucun bon sens de vacciner les enfants de 5 ans de toute façon. Le virus est rendu scrap, il ne fait plus rien. Ce n’est plus comme il était avant, il y a eu trop de mutation. Puis quand il mute, un virus, bien il est moins mortel. Je ne le sais pas, en fait, je ne le sais vraiment pas qu’est-ce qu’on faire.

**Je suis tout à fait d’accord. Puis je comprends entièrement ces hésitations-là. Et est-ce que vous êtes plus sensible quand un professionnel de la santé va exprimer des doutes sur la COVID? Si admettons vous voyez un témoignage d’un professionnel de la santé qui remettait en question la vaccination contre la rougeole?**

003 : Bien là ça serait déjà fait.

001 : C’est prouvé depuis… Comme [003] le disait, ça fait longtemps qu’on le donne. Si lui se met à douter, peut-être. Il y en a quand même plusieurs qui mettent en doute le vaccin, de comment il est administré. Je l’ai fait quand même. Ça reste plus inquiétant, oui, que si c’est mon ami qu’il est contre. Là, quand je vois un médecin qui remet en doute, c’est vrai que ça inquiète.

003 : Oui, mais tu sais, c’est ça le problème, si un médecin met ça en doute, on ne l’entendra pas, il va être muselé. Jamais, on ne va l’entendre. C’est ça le problème.

**C’est sûr que c’est difficile dans le contexte actuel d’avoir une position nuancée. Souvent c’est un peu… les gens sont très pros, très contre. Puis là ceux qui sont très contre, bien ça va à l’encontre du Collège des médecins et tout ça, puis là évidemment, il y a des plaintes. Bien on a des exemples en tête au Québec.**

**Mais bon, revenons à nos moutons et notre histoire. Je comprends que comparativement, disons à la première histoire, celle avec le professionnel vous rejoint plus, vous interpelle plus…**

[Tous les participants font le signe ‘’oui’’ de la tête]

**… , c’est quelque chose que vous écouteriez jusqu’au bout, si vous n’étiez pas dans le contexte d’un groupe de discussion, peut-être?**

001,002,003 : Oui.

**Je propose qu’on passe à la troisième histoire, puis après on aura une discussion générale.**

**Histoire #3**

*« Une mère très hésitante à la vaccination était convaincue que les professionnels de la santé ne disaient pas la vérité sur les vaccins. Son enfant a finalement attrapé la coqueluche et a subi de graves complications. Sa vision du vaccin et du personnel de la santé a fortement changé ; elle comprend qu’ils ne désire que la santé et la sécurité des gens».*

**Celui-là est en français aussi.**

**Visionnement du vidéo #3** [témoignage maman et enfant de 30:54 à 34:37]

**Donc j’aimerais avoir vos premières impressions sur ce récit-là. Évidemment on change de registre avec cette histoire-là.**

002 : Ça m’a vraiment touché, j’ai failli pleurer dans le vidéo. C’est vraiment venu me chercher comme en tant que parent, mère ou père, dès qu’on voit un enfant qui a été malade ou quelque chose comme ça, ça vient vraiment nous chercher. Donc pour moi, ça m’a vraiment impacter.

003 : Oui, ça scrape un peu. Je suis comme un peu frustré en même temps, mais combien d’enfants… C’est quoi le pourcentage qui vont virer de même? Mais méningite, je pense que mes enfants ne l’ont même pas eu, le vaccin. Puis d’habitude c’est dans la liste de vaccins, mais il me semble qu’il n’est pas là.

001 : Non.

002 : Moi j’ai envie d’aller regarder…. Après ça, j’étais comme : «ok je vais aller voir le carnet pour m’assurer qu’elle l’a reçu». En fait, je ne sais même pas... si elle l’a reçu, est-ce qu’on pourrait juste lui redonner juste au cas où, parce que je ne voudrais vraiment pas que ça arrive. En tout cas, pour moi, c’est venu me chercher.

003 : Moi aussi.

001 : Oui, moi aussi, je suis un peu sans mot. Puis ça se traite en même temps. Parce que je pense que c’est un vaccin que tu vas chercher à part, je suis comme tombée là-dessus en faisant une recherche sur internet. Mais j’ai une amie que c’est arrivé à sa petite fille de même pas 6 mois, donc elle aussi c’était entre la vie et la mort, puis finalement elle est correcte. Puis tu vois, je lui ai envoyé le lien du vaccin, puis elle m’a dit qu’elle n’en avait jamais entendu parler. Donc même en ayant sa fille qui a eu ça… Mais c’est parce qu’il y a des types de méningites, donc je ne le sais pas si c’était une de celles-là. Je ne sais pas si c’est celle-là en particulier qu’elle a eue. Bien elle a eu celle qui était grave, ça doit être la même que le petit garçon. Mais oui, c’est inquiétant, donc c’est sûr que je vais m’arranger pour faire vacciner ma fille, pour ne pas prendre de chance. S’il y en a un qui existe, puis qui est prouvé, je ne vois pas pourquoi je ne le ferais pas.

003 : En plus dernièrement… Bien en tout cas, la plupart du temps ils disent: « si ton enfant... il faut que tu attendes trois jours de fièvre avant de l’apporter à l’hôpital». Trois jours de fièvre, c’est ça qui arrive, il est trop tard si c’est une méningite. Moi, j’en ai eu une dernièrement qui avait 3 jours de fièvre puis c’était une pneumonie, je pense qu’on a été chanceux. Mais si ça avait été ça, ça aurait mal viré.

**C’est effectivement très fulgurant des méningites. Au Québec on vaccine contre le méningocoque C qui est le plus commun, à 12 mois. Donc il y a un vaccin qui est dans le programme. Ce type de méningite là que ce petit garçon-là a eu, il y a un vaccin aussi. Il a été offert dans une campagne ciblée quand il y a des éclosions. Donc ça a été offert au Saguenay-Lac-Saint-Jean parce qu’il y avait eu plusieurs cas.**

003 : J’en ai fait partie justement. Ils nous ont vaccinés quand j’avais 19 ans, je pense. Ils ont vacciné tout le monde, au complet. Il n’y avait pas vraiment de cas. Je ne sais pas pourquoi ils nous ont vaccinés comme ça, mais bon, on l’a tous fait.

**Il y a de grandes campagnes, puis c’est sûr que ça demeure quand même très très rare, des histoires aussi tragiques. C’est sûr que dans l’histoire #2, le professionnel de la santé parlait d’un enfant qui est décédé, mais j’imagine que ça n’a pas le même impact que quand ça vient d’un parent, puis qu’on voit aussi l’enfant avec toutes les séquelles.**

003 : Oui, c’est dur à regarder.

001 : Oui.

**Puis généralement des histoires qu’en santé publique on évite d’utiliser parce que ça joue sur une émotion très forte, mais vous, dans une stratégie pour rejoindre des parents qui seraient plus incertains, est-ce que vous trouvez que ça serait une bonne stratégie?**

003 : Non.

002 : Je trouve que oui, c’est très efficace. Parce que justement des fois… Premièrement, on n’est pas au courant que ce vaccin-là existe. Deuxièmement on ne voit pas les impacts que cette maladie pourrait avoir sur nos enfants. Donc de voir ça, jusqu’à quel point. Puis là, on se dit:« bien ça pourrait m’arriver. Je pourrais être dans cette situation-là. Ma fille pourrait être dans cette situation-là. Je ne voudrais vraiment pas la mettre dans cette situation». Donc moi je trouve que c’est très efficace. Mais oui, c’est vrai que ça joue sur les émotions.

001 : Mais en même temps je trouve que c’est… Ça joue sur les émotions énormément, ce n’est pas un vidéo qui est facile à regarder. Peut-être que moins intense que celui-là, mais en même temps ça a l’effet escompté. Je vais donner l’exemple de l’annonce du cancer, les gens qui revolent en arrière, c’est quand même la réalité de cette maladie-là, ce que ça peut faire. Donc je trouve ça important de le monter. Parce que comme [002] le dit, on ne voit pas nécessairement les impacts, ou on a souvent l’idée que ça arrive aux autres, mais pas à nous. Mais on dirait qu’en le voyant c’est encore plus… La madame, c’est une madame comme tout le monde, donc ça pourrait nous arriver à nous aussi.

003 : Moi je pense que de faire une publicité avec ça, il y a une limite à ne pas passer. C’est comme les accidents de la route ou l’alcool au volant, des choses comme ça. Il y a une limite où est-ce qu’il ne faut pas que tu passes... Tout ce que tu fais, c’est faire peur à ta population pour avoir le but que tu veux en tant que gouvernement. Puis il ne faut pas aller ça. Il y aura toujours du monde qui va dire non aux vaccins. Même s’ils voient ça, ça va leur donner du gaz pour ne pas le faire. Je ne sais pas comment ils réfléchissent, mais… Je ne sais pas quelle sorte de publicité pourrait les convaincre non plus. Mais ceux qui ne sont pas convaincus …

002: [INAUDIBLE 42:02] je trouve pour convaincre quelqu’un.

003 : Ils sont inconvaincables. C’est le 5% comme on appelle.

002 : Ça dépend de la raison, pour quoi ils ne veulent pas faire vacciner.

003 : Oui, mais rendu là, je pense que c’est rendu de la religion ou des choses comme ça.

002 : Bien juste dans le vidéo, la raison pourquoi la madame… C’est parce que ce n’était pas disponible. L’autre, elle n’en avait pas entendu parler. Donc il y a comme un manque d’information, pas juste les gens anti-vax, complotistes comme on le voit trop souvent en ce moment. Il y a des gens aussi qui ne le savent juste pas. Ils ne sont pas informés. Ils sont peut-être moins éduqués. Donc ils n’ont pas accès à l’information. Ils ne connaissent pas leurs ressources. Ils ne vont pas aller voir le médecin, les CLSC et tout ça. Donc cette partie-là peut-être pourrait… Ça pourrait être efficace pour ce monde-là, mais je comprends que…

003 : Oui, mais juste donner de l’info pour dire:« hey ce vaccin-là existe». Tu n’es pas obligé de mettre une affaire qui fesse comme ça. Juste une info du genre : «hey, méningite, pensez-y parce qu’il n’est pas dans la liste des vaccins obligatoires». En tout cas, moi, je ne sais même pas c’est quoi la liste . On est allé au CLSC, ils ont vacciné nos enfants, puis that’s it, je n’ai pas... Il faudrait que je check. C’est sûr que l’hépatite B n’est pas dedans. Il n’y en a pas.

**Bien il y est donné aux élèves en 4^e^ année scolaire, hépatites A et B.**

003 : Ah oui?

**Oui. Puis maintenant il est inclus dans le vaccin hexavalent. Donc il y a une dose avec polio, diphtérie, coqueluche,**

003 : Oui je sais, il y a des doubles… Je ne savais pas...

**C’est un combo.**

003 : Hey bien, on en apprend!

**Mais je comprends qu’il y a des positions divergentes. Donc c’est bien ça touche, ça appelle aux émotions. Mais en même temps, ça pourrait peut-être chez certains parents très hésitants, les braquer encore plus, ou les rendre… Est-ce que vous trouvez que de jouer sur la peur…**

003 : C’est jouer sur la peur et les émotions. En tout cas, je pense qu’il ne faudrait pas aller là. [Le participant 003 doit s’absenter quelques minutes].

002 : Oui, mais quand il y a un danger réel, il faut connaître la vérité aussi sur les dangers potentiels. Tu dis : «faites-vous vacciner contre la méningite, point». Pas nécessairement. Par contre, tu dis: «Va te faire vacciner contre la méningite parce qu’il pourrait avoir telle et telle, telle chose, les effets pouvant causer la mort», là peut-être que les gens diraient : «oh!, OK». Donc moi je trouve que c’est important de faire face à la réalité.

001 : oui moi aussi je trouve c’est [INAUDIBLE 45:06]…

**Puis est-ce que vous oseriez partager ce vidéo-là à une amie ou à un autre parent qui aurait des questions ou des doutes sur la vaccination en disant : «regarde ça...», ou vous seriez un petit peu moins…?**

001 : Bien ce n’est pas mon opinion à donner si elle ne veut pas le faire, sauf si elle me questionne, oui je vais le faire. Je lui enverrais, moi je ne suis pas non plus en campagne de vaccination. C’est sûr que si elle me demande mon avis, je vais lui en parler. Puis moi ça me touche dans le sens que comme je l’expliquais, j’ai une amie à qui c’est arrivé également. Puis tu vois, ça m’a affecté de savoir que quelqu’un de proche à qui c’est arrivé de savoir que sa fille avait peut-être 24 heures entre vie et la mort, c’est arrivé à quelqu’un proche de moi, donc moi ça m’a encore plus interpellé. Je veux dire de voir ce vidéo-là pour des gens qui n’ont jamais eu d’amis proche, je trouve que c’est quand même… ça appelle à la vaccination.

002 : Si quelqu’un me questionne oui, je serais confortable à montrer ce vidéo-là, mais pas si la personne a ses opinions… C’est parce que des fois les parents, c’est vraiment *touchy* de donner son opinion. Des fois on reçoit trop d’opinion : «fais-ci, fais ça, fais-ci, fais ça», donc ça pourrait justement causer l’effet contraire.

001 : Oui.

**Donc si je vous demande dans les 3 vidéos ou les 3 scénarios qu’on vous a présentés, lequel vous préférez, puis lequel vous aimez le moins?**

001 : Moi je dirais sûrement celui du professionnel que j’aime le plus. Celui qui a le plus d’impact par contre, c’est celui de la maman. Ah, je ne le sais pas, c’est lequel que j’aime le moins, parce que dans un sens celui de la recherche internet, oui me je suis quelqu’un qui sait qu’il y a de la désinformation, mais ce n’est pas tout le monde. Il y en a beaucoup de personnes pour qui c’est dur comme fer… Je ne sais pas lequel que je pourrais vous dire… Personnellement moi, oui c’est sûr que c’est celui du professionnel, j’y vais avec le personnel. Mais je trouve que les 3 sont quand même bons, dans le sens que ça peut toucher toutes les personnes comme ceux qui regardent juste sur internet, puis qui font leur propre idée, parce qu’il y en a.

003 : Je suis un peu d’accord avec [001]. Le vidéo du professionnel, je pense que c’est lui qui aurait le meilleur impact. L’autre que j’aime le moins c’est… bien vous l’aurez deviné c’est le flô qui n’a plus de jambe, plus rien, lui, il passe trop à l’extrême. C’est quasiment… Même si tu as une méningite, tu ne vires pas comme ça tout le temps.

001 : Non.

003 : Il montre la pire patente au monde. Puis là le monde va dire : « voyons donc...». Je sais que ça va virer… Il va y avoir des sacrages, puis des affaires de même.

002 :Moi aussi, je suis d’accord avec [001] donc celui qui me rejoint moi personnellement, c’est celui du professionnel. Justement comme elle a dit, celui qui a le plus impact potentiel serait le dernier vidéo. Mais aussi comme elle a dit, le premier a aussi un impact, pas pour moi, personnellement, mais pour d’autres personnes, parce que je sais qu’il y a une grosse majorité des gens qui prennent leurs informations sur internet. Donc ça pourrait aller rejoindre ça. Mais pour moi, personnellement, pas vraiment. Ça serait le deuxième, puis après ça le troisième.

**Puis généralement, est-ce que vous trouvez que l’information sur la vaccination dans les vidéos est nuancée? Elle vous permet de prendre une décision, ou non il manquerait quelque chose pour vous aider, un petit complément pour que vous puissiez prendre une bonne décision? Ou, vous trouvez que c’est trop pro ou trop extrême?**

001 : Bien si je me fies au vidéo du professionnel qui me touche plus, j’aimerais quand même… Je ne sais pas comment le dire, mais savoir l’effet du vaccin dans notre corps, aussi. Pas juste admettons:« c’est bon, j’ai déjà vécu ça, c’est bon, les enfants l’ont moins», je veux un peu savoir, si oui j’ai fait vacciner ma fille, mais en tant que tel qu’est-ce qu’ils mettent dedans? Je parle juste s’il pouvait ajouter quelque chose, ce serait ça, même si je me fies quand même à l’effet du professionnel.

003 : Plus un.

002 : Moi aussi je trouve que ce serait une bonne idée d’avoir justement une des craintes majeures, admettons que ce serait les effets secondaires du vaccin qu’un professionnel réponde justement dans le vidéo à cette crainte majoritaire-là des gens qui sont justement réticents à se faire vacciner. Le troisième, c’est sûr que c’est vraiment provaccin. Je trouve que le deuxième est quand même plus équilibré. Puis le premier est vraiment beaucoup plus neutre. Donc il n’est pas assez peut-être pour la vaccination. Il dit juste:« va chercher tes informations», mais on ne parle pas vraiment… C’est vraiment plus général.

003 : Vous m’avez fait penser à quelque chose. J’aimerais ça aussi… Un certain vaccin pour une certaine maladie change. Parce que là ils sont en train d’injecter du ARN-messager à tout le monde, est-ce qu’ils vont changer les autres vaccins, pour la même technologie? Est-ce qu’ils vont nous le dire? Puis ça, je suis sûr qu’ils ne le diront pas. Parce qu’ils disent : «ah c’est le vaccin pour la rougeole», est-ce qu’ils l’ont changé? Moi je voudrais avoir l’ancien vaccin parce qu’il marche, je ne veux pas qu’ils le changent. Puis ça c’est quelque chose que j’ai beaucoup de crainte en fait, pour savoir s’ils vont changer les vaccins, puis la technologie là-dessus?

**C’est une question légitime. C’est sûr que normalement dans le consentement éclairé on a le nom générique du vaccin, puis la technologie. Pour l’instant tous les autres vaccins actuels ont des plateformes, ils sont bien développés. Ils sont produits, donc ça serait surprenant. Mais effectivement, c’est une question légitime. Est-ce que si dans nos histoires on fournissait des types d’informations sur par exemple Santé-Canada, ce serait quoi les sources qui vous interpelleraient le plus?**

003 : En fait le best ça serait… Bon Santé-Canada slash vaccin, puis là tu as toute la liste depuis quand il est injecté et puis les effets secondaires qui vont avec. Tout simplement, juste ça.

**Je pense que ça existe, puis tu as les dates, les compositions des vaccins, les ingrédients. Mais pour trouver de l’information sur le site de Santé-Canada, ça prend un diplôme en archéologie.**

003 : Exactement, oui (rire).

**Ce n’est pas toujours très facile à trouver. Quand on va concevoir nos histoires, on se demandait… Là pour l’instant les 3 vidéos ce sont de vraies personnes, donc ce sont vraiment des parents et des professionnels de la santé et une maman. Est-ce que pour vous c’est préférable que ça soit vraiment une vraie histoire ou si vous pouviez ressentir la même chose ou vivre la même émotion si c’était, disons des images avec une voix ou un style cartoon. Bien on s’entend que pour le dernier, le témoignage de la maman, c’est peut-être moins percutant.**

002 : Moi je préfère des personnes réelles, de vraies histoires, de vrais professionnels, de vraies mamans puis des papas.

003 : Oui, moi aussi.

**Puis le médecin qu’on a vu c’est un professionnel de la santé qui pratique au Québec, mais qui est d’origine française, est-ce que ça vous a agacé qu’il ait un accent? Est-ce que vous auriez préféré…?**

003 : Non, vraiment pas (rire).

001 : Bien non, un professionnel, c’est un professionnel, peu importe.

003 : J’ai plus confiance à ceux qui viennent de la France que ceux qui viennent de Santé-Canada. J’ai comme une idée un peu péjorative de Santé-Canada qui sont juste des pouceux de crayons qui suivent l’organisme des États-Unis.

001 : Ah, oui. Je comprends.

003 : Je ne me souviens plus c’est quoi. La FDA, c’est ça.

**Parce que par exemple, la dernière maman, c’est clairement une maman québécoise qui avait un accent québécois, donc est-ce que vous connectez plus avec quelqu’un ou pas nécessairement?**

003 : Moi, ça n’a aucun effet. Je ne suis vraiment pas une personne générale. Je suis allé travailler en Europe 3 ans. Pour moi, il y aucune différence.

001 : Non, moi non plus, ça ne change pas. C’est un professionnel comme je le disais tantôt, donc un professionnel, ça reste un professionnel, peu importe, il n’est pas obligé d’avoir un accent québécois pour avoir un impact.

002 : Oui, moi aussi.

003 : Oui, mais je pense qu’il y en a certains qui vont…

001 : Oui c’est ça, mais il y en a que oui… Mais moi, personnellement non.

003 : Mais c’est sûr qu’il y en a plein. Même une personne de couleur ou un asiatique ou n’importe quoi qui a un accent, ça ne pognera pas sur certaines personnes, ça, c’est sûr. Au Québec, on est encore là, c’est plate, mais c’est ça.

**Puis pour les plateformes. Vous avez tous dit que vous utilisez Facebook, si on voulait diffuser ça en ligne, pas quelque chose qu’on voudrait faire passer à la télé, mais vraiment une intervention qu’on voudrait faire dans les médias sociaux, ce serait quoi le véhicule pour rejoindre le plus grand nombre de parents? TikTok est-ce que c’est quelque chose que vous regardez?**

[Les participants 002 et 003 font le signe ‘’ non’’ de la tête]

001 : Bien pour les parents ce serait Facebook. Tiktok, c’est pas mal les jeunes. Les plus jeunes ou sinon à partir de mon âge. Mais Facebook rejoint un plus monde. Il y a aussi peut-être Reddit, je pense, qui est quand même assez regardé. Ceux qui sont sur Facebook vont sûrement sur Reddit.

003 : Même déjà que ça soit Facebook, ils ne me rejoindront pas, mon compte est bloqué. J’ai 40 amis, puis je trouve que j’en ai trop déjà. Donc je ne le verrai jamais l’annonce. Je ne la verrai pas. Donc le best ce serait une annonce à Radio-Canada tout simplement, une annonce pas très longue, mais qui a un site internet très très petit où est-ce que le monde peut aller voir. Genre : «informez-vous [INAUDIBLE 57:39] slash vaccin» , quelque chose du genre. Je pense que ça serait le best. Parce que c’est sûr que Facebook, il y a aucune publicité qui entre dans mon compte.

**Parfait, donc tu ne vas pas cliquer sur tous les trucs qu’ils mettent sur le côté de la page pour essayer d’influencer des achats.**

003 : Non, je l’ai enlevé.

002 : Moi c’est parce que je ne connais pas trop les réseaux sociaux, donc je dirais Facebook de ceux que je connais. Mais comme je n’en connais pas vraiment, ce serait plus comme à la télévision comme une publicité traditionnelle.

**Je regarde mes questions pour voir si j’ai fait le tour. Bien oui, j’ai posé toutes les questions. Marie-Eve, Dominique, Laurie-Anne si j’oublie des aspects, peut-être ouvrir vos micros puis faites-en part. Est-ce que vous aviez d’autres commentaires, suggestions, stratégies? Je comprends que oui les histoires… Si je résume, oui les histoires ça touche, ça peut comme susciter un intérêt, mais selon vous, ça prend quand même un autre complément d’information peut-être plus détaillé ou spécifique pour accompagné ça pour prendre une décision ou pour vous sentir plus confiant dans des décisions que vous prenez, par exemple?**

001 : Oui.

**[003] tu réfléchis, as-tu des…?**

003 : En fait, vous le but, c’est de trouver une manière de rejoindre les gens pour les convaincre d’aller se faire vacciner, c’est ça?

**Effectivement. Bien en fait le projet prend sa source de quand on regarde des contenus contre les vaccins ou des critiques envers les vaccins, il y a beaucoup d’histoires personnelles qui sont partagées. Donc on va partager beaucoup d’histoires sur les effets secondaires, puis c’est pour faire un peu un contrepoids à ces histoires-là.**

003 : Bien aujourd’hui je te dirais, les jeunes sont tout le temps, tout le temps sur les affaires qui sont ultrarapides donc quelque chose qui est trop long, ils vont décrocher. Donc moi ce que je ferais c’est : tu vois quelqu’un qui est contre le vaccin X, puis tu vois quelqu’un qui est pogné avec la maladie X, mais assez rapide. Un autre qui est contre le vaccin Y, puis l’autre qui est pogné avec la maladie Y. Puis là, tu l’envoies en chaîne comme ça. Mais pas d’avoir d’émotion comme on a vu avec la madame, ça, c’est trop, parce que c’est trop long, puis là tu viens à t’attacher à elle, puis tout, puis c’est juste pour une maladie. Donc moi j’irais comme en chaîne comme ça. Genre cligne, cligne, cligne, cligne, cligne, tu as 20 secondes, tu peux en passer quasiment 5. Tu as une idée générale:« le vaccin, ce n’est pas pour rien», ou quelque chose du genre. En plus, ça rime.

**Avec le slogan, tout.**

001 : Il va la faire (rire).

003 : J’envoyais ça de même. Je verrais ça comme ça, puis là oui j’accrocherais. Parce qu’il y aurait plusieurs vaccins, «informez-vous»…

**Court. Donc déjà, le format comme on vous l’a présenté 2-3 minutes, c’est comme un peu trop long?**

003 : Oui, c’est trop long.

002 : Mais tu sais, moi la deuxième étais moins longue. La première était longue. Je l’ai trouvé longue. La troisième aussi. Mais la deuxième, il me semble avec le professionnel n’était pas si longue que ça.

**Elle est un petit peu plus courte. Mais on n’est pas dans le clip de 20-30 secondes.**

003 : C’est sûr que ce ne sont pas des clips. Mais aujourd’hui, je suis pas mal sûr que ce qui va rejoindre les gens, ça va être des clip un par un.

001 : C’est sûr que ça doit être court parce que c’est vrai qu’aujourd’hui on est très dans l’expéditif, donc tout ce qui est trop long… Même les vidéos qui défilent devant toi, si tu n’as pas l’information vite, on finit par changer.

003 : Puis de quoi aussi, que tu n’as pas besoin d’avoir de son. Pour accrocher des gens, quelque chose que le monde va comprendre sans avoir le son. Parce que moi dès qu’il y a une annonce, je mets mute parce qu’ils augmentent le son. Puis je ne veux pas réveiller les enfants.

**C’est vrai que c’est un bon point. Puis pour les gens qui regardent leur téléphone dans les transports en commun, effectivement.**

001 : Oui, c’est bien ça.

**D’autres informations, commentaires avant qu’on termine la rencontre?**

001 : Moi ça fait pas mal le tour.

**Focus group 4**

**Thanks. So, the goal of tonight's discussion is really... we're testing intervention to promote childhood vaccination in general. So, we want your feedback on these interventions. We want your frank comments. So, if there's things that you don't like or you think are not working, don't be shy about telling us. We won't be angry at you. It's really the goal to [INAUDIBLE4:01] these interventions before testing them on a larger scale. But before getting into the meat of the discussion, if I say the word ‘’vaccine’’, what comes to your mind, first?**

##### [0](https://www.happyscribe.com/transcriptions/dfe5b08d2f7e47a18143073200760acd/edit_v2?position=256.95&utm_source=happyscribe&utm_medium=document_deep_link&utm_campaign=export_docx&utm_content=dfe5b08d2f7e47a18143073200760acd)04: It's a hard question (laugh) because it's general, it’s like...

001: Like flu shots and stuff like that, very general kind of vaccines.

##### [0](https://www.happyscribe.com/transcriptions/dfe5b08d2f7e47a18143073200760acd/edit_v2?position=273.85&utm_source=happyscribe&utm_medium=document_deep_link&utm_campaign=export_docx&utm_content=dfe5b08d2f7e47a18143073200760acd)02: In the last two years, I guess we can almost say now : ‘’it's a way of life’’, a way to move forward. Almost an expectation, I guess, depending on what you want to do, where you want to go.

##### [0](https://www.happyscribe.com/transcriptions/dfe5b08d2f7e47a18143073200760acd/edit_v2?position=291.23&utm_source=happyscribe&utm_medium=document_deep_link&utm_campaign=export_docx&utm_content=dfe5b08d2f7e47a18143073200760acd)03: Yeah, it's like they asked in schools, all the time. So it's kind of a thing that most kids need when to go to school.

**Not in Quebec, but in other province. Okay, so, we're developing stories, we're using video stories or text stories, we're not quite sure yet, to promote vaccination. We often hear people sharing their experience about vaccinations. So, we want to do the same, but to talk positively about vaccinations. So, we have three stories that they will be shown to you and we will discuss each of them. And then, have a general discussion on the three. So, do you have any questions or concerns before we get started?**

002: Not yet.

**Okay, great. So Marie-Eve, if you can share your PowerPoint. So, we have kind of the story [INAUDIBLE6:11]. And then, we'll show you a video that would look like the final story that we will test. So the first story is...**

**Story #1**

**A medical doctor that explains his experience with the tragic death of a child that could have been prevented by vaccine should that parents have known and accepted the vaccine. So, the idea is a healthcare provider talking about his experience with vaccine preventable diseases. And we have a short video to show you what this would look like. It's a bit old, so don't look at the picture or the dressing, but it's an idea of the story we want to discuss.**

##### *[ Léger bogue informatique]*

**Viewing video# 1 [Hib vaccine - doctor at 8:52 until 9:37 ]**

**So, what are your first impression of that video? We know thing that it's a bit old, but otherwise...?**

##### [0](https://www.happyscribe.com/transcriptions/dfe5b08d2f7e47a18143073200760acd/edit_v2?position=588.09&utm_source=happyscribe&utm_medium=document_deep_link&utm_campaign=export_docx&utm_content=dfe5b08d2f7e47a18143073200760acd)02: Relax (9:48) content. I find when you see videos and stories like that, if they show lives examples some strikes me when you see kids... sick kids in hospital. It’s kind of hits maybe a bit more. Maybe pay more intention to what's be broadcast versus just a doctor, just speeking on his own.

004: It’s reminds me one more time that prevention is better then treatment. They have to really insist on this point. Even if we know that it could have had some side-effects. But, it still ringing on my bells, that’s always what we always say ‘’prevention is better then cure or treatments’’.

003: During the video, we doubles felt like the doctors is more like speaking to other doctor. I think I am sharing his opinion and his perspectives, and how it impacted him. If you focus more how it impacted him as a doctor, as a human being, as opposed to just... It felt really like directed almost charter fellow doctors 11:25], I suppose, to the general population.

**Because it was to complex or because he was not speaking about his experience of treating those children?**

003: It’s just felt kind of impersonal, almost like a warning to doctors. It was not steering much emotion. I think what we seeing, it is super important, but it don’t fell emotional. It felt like acted. Like, I mean, he is an actor.

001: I think it would better if we had a different angles of camera shots or if we bring some graphs for the statistics that he said. The way I see it, it’s been shot... the advertise might been shot on one single take. And it’s much less interesting and attractive as oppose to... if they have shot it with different angles. And, perhaps with even children.

**And did you felt that the information about vaccine was balanced or nuanced enough? That’s represent both risk and benefits of vaccine?**

001: I think it did present the benefice and the risk, somewhat. In the beginning, I was more incline to listen what he has to say. And towards the end, my attention just ran away. (laugh). I don’t know, maybe it’s because of his tonal voice and all the things. So many things he has to say...

**Any others opinions on the balance of information, or the level of information. I know [003] you suggested that it’s was not enough or that it could be have graph to show other aspect.**

003: I think what [001] said is really accurate, I really felt it nailed there. He had the word that I couldn’t get.

004:It’s mostly alertly, than emotional.

**And what do you think is the key message, for that story?**

003:What he said right now, or what he should say?

**What do you think it was the message: ‘’go to get vaccine right away’’...**

003: If it was like ‘’if you don’t get vaccines, you can get really injured’’ [INAUDIBLE 14:50]

004: Yeah.

002: Almost like air of caution. More caution, and not really being speaking more prevention at all. It was more a caution approach.

001: He is saying we are paying less attention, we are giving less attention to the hits vaccine, and people should be more aware of it, and it’s there to protect children. He try to encouraged parents to have their kids, to get their vaccine. But it’s not like convincing (laugh).

**And do you feel that if it was coming from a parents it will be better then from a healthcare provider? What do you think of the fact this is a physician speaking?**

[Nobody answer]

**Is it more credible?**

001: First, he is not dress as a physician, as a doctor. He looks like an average person setting in a park and doing his talking. He looks less credible the way he’s dress with the background.

**Maybe a white coat...**

001: yeah, a white coat, in a clinic, doing this speech would be seem more credible, to me.

002: A medical setting may have been a bit more appropriate, more maybe... eye opening.

**And would that have the same impact if that message was by a nurse, for instance? Or do you think physician are the most credible to speak about vaccine?**

003: I think with him discussing his experience and what he said, I think from a nurse... Considering the thing during COVID and how they talk a lot about how the nurse was in front line, having come from a nurse after all that, it would just go alone what we have been hearing a lot, nurses are in the front line to see the pretty and the not so pretty. Because his sharing about how it’s not fun to see that. Even if it was more medical setting, like everybody is saying, it give more credibility with it. More power behind this work, for sure.

004: To me also, it doesn't matter if it's a nurse or doctor or if it's public authority, it doesn't matter. The most important thing to me, it's like speaking on behalf of the authority would give them more credibility, not only like a vegetable. Like I come to sit in the park and say ‘’hey, if they haven't done this(18:32¼’’. I think when the slogan comes with the authority like public authority or some places like that. And logos of them, for example Health Canada or something like that, it going to give them more credibility, then no matter if they are doctor or nurses.

003: Finishing it off almost with ‘’this message was brought to you by the order of physician of Quebec’’, or something like that.

**And what type of information could be added in this such type of a video, to make it better, just to provide more information? Would you like to have statistics on diseases, statistics on risks of vaccine, vaccine efficacy? Or, that's not important?**

002: I think it would be great. I think one that strikes a lot there, you starting to see now with this new variant that everyone is talking about. Talking the rates of infection, the rates of hospitalization versus vaccinated; 23 times higher versus if you're not vaccinated. So, I think numbers putting quick fact numbers. Keeping the message easy. Easy to follow. Right in your face, type thing. And then, another thing I would touch on two, is we all know how fragile the healthcare system is... or, has been and probably continues to be right. So, maybe just putting a bit of an emphasis on that as well. Kind of talking, saying... I don't know like thinking from a nurses perspective and a doctor, everyone is tired. It could be another thing, maybe just a quick pointer like ‘’ think about us’’ in a sense. I don't know, maybe like ‘’ we're tired, here's some info’’. I don't know. Just to put some facts in their face and just say...

**And having a logo of Health Canada or the Quebec government, for some parents, that's a drawback. They don't trust the authority and they don't like that, is it the same for you?**

004: No, not for me, honestly. Because there are the ones who are certifying you, or certifying the doctors or the others. I personally believe, if I don't trust them, how can I trust the others who are trusted by them.

001: I would put more trust in both... If my family doctor recommends it. In another same time, if either the government of Quebec or the federal government recommends it. So, I'll take my decision on those things.

003: I think if it's coming from a doctor like... If we were talking about a video that came from parents, maybe you wouldn't want to put the logo necessarily, because you're kind of... like targeting more. But when you bring the doctor, you're bringing in the authority. Like [INAUDIBLE 21:51] make it clear that's what you're going to do. Because that's kind of what you're aiming to do with the doctor...

**Great, thanks. And before we move on to the second story, just for you to know [INAUDIBLE22:08] is a world leader vaccine researcher. He developed himself some vaccines in his labs. So, maybe those who develop that video just thought ‘’oh it's so much well known by those of us, who do vaccine research, that everyone will know him. No need of white coat and to put’’, so that might be...**

004: Yeah, to make it more friendly, maybe.

**Yeah, make it more accessible, like a real person. I don't know Marie-Eve, if I'm just better to go on with no slides and you can only share the second video?**

***I think the PowerPoint is working now. Maybe I can try to share it.***

**So, the second story we want to test is really about how parents make decisions about vaccines. So, it's really about a mother or father were not sure about vaccinating his or her kids, and want to seek information. And he is overwhelmed by the quantity of information online. Don't know where to find the right information. Don't know which information to trust. And then, by searching on government websites and by talking to others, they finally make the decision to vaccinate. But it's really about the process of making a decision, or making an informed decision, and how to navigate the Internet to find good information source and avoid be too worried by false information, or fake news about vaccines that are online. So, that's the gist of the second story and I'll show you the video.**

***Viewing video #2 [parents hesitating about vaccines 24:13 at 27:41]***

**So, first impressions of this other video?**

003: My wife saying that she liked it a little more than the other one. Having a lot of... actual people telling their story had a bigger impact for her, she says.

**Great, and for you?**

003: I found it long.(laugh) It was long. It was good. I wish it highlighted a bit... At one point, they showed stats about... But, it was so small. I don't really have time to really read it. If you're going to show me something, put it in my face. I want to work for it... I'll go and research myself. If you're showing me a video, show me the stats. That was my big takeaway. It was long but it was good. The story was good and it was really interesting.

002: I guess the reality work, even now... A grown up kind of knowing and seeing vaccine and the efficacy and the importance of being vaccinated. As you said, certain provinces here like we are just across from Ottawa, we have friends who are kind of (INAUDIBLE29:15] in that system who have to be vaccinated, their kids have to be vaccinated to go to school and all that. So, it's more of an expectation around here, I would say. And it's not as taboo as one would think. I agree. I guess I agree with the doctors, like if you're going into a public school out there, you want... if you take the efforts to vaccinate your children, then you want to kind have that cascaded across the network type thing. But I think the information was good. As [003] was saying, they could have added more statistics, for sure. And focused a bit more on that, but overall, it was good.

**Thanks, maybe [001], and then [004].**

001: I think it's a very good story that explained how the two parents from the beginning they didn't vaccine the first child. And with the research, I'm not sure what kind of research they did in the first time or what kind of website they went to, in order to get the conclusion to not have the first child vaccinated. But it seems that... with the second child, they had time, I guess. Obviously, they took longer more time to research and to have their own opinion to form their own conclusion about the benefits which outweighs the inconvenience of the vaccine. So, we see that as parents. They also grow in the knowledge because with the first kid, they had... maybe back then, the Internet had less correct information circulating on the Internet back. I'm not sure what year that was. But now, we have many websites, official websites like the WHO that they mentioned, that are the authority of vaccines. So, people are able to get more accurate information from those websites and draw their own conclusions based on researches not on the different test results or that(31:53).

**Good. [004], anything else to add?**

004: Ah no, actually I'm at the same point as them. It was very better than the first one. But first, it was long. Second, the message transferred to us was really blurriest. Like, finally, she was satisfied or he was satisfied. She said ‘’ I would have done differently’’, but she didn't mention how, what it was missing at this point? Honestly, I didn't get the point. Finally, it was good or not? So, it's like too much teams(32:36) or too much information, but without not to the point, without a clear message.

**That's interesting. So, the key message is unclear?**

004: Exactly.

**... what they’re trying to**

004: What was the main message? Or, what is the main slogan of this video, at the end?

**What do you think is missing in that video to make it better?**

004: Make it shorter, but to the point. Uh, if they show us this statistics, it was really quick. As [003] said, it should be on our face, to see what they have seen. Where they have referred to? And how she would have done that differently? Because she said ‘’I would have done this differently then now’’, so... I don’t know honestly the point (33:35). What was missing? Exactly, this my question : ‘’What was missing, at that time?’’, ‘’What made you interested more?’’. ‘’And how could you have the made it different, from the other... prime?’’.

**Do you related to these parents? Can you identified with them?**

004: Somehow.

002: Yeah, for sure, I can identified. With this vaccine for children that's coming on now, do I want to get my children vaccinated? Absolutely. But, right away? Maybe not. There is so many unknowns. I can’t want to see some data, maybe see some facts, see how things react. Before lining up on net(34:33). I think a lot of people are in that same boat, so I can definitely related to the parents, hesitations on that front, and waiting to see some fact based information, before going to that route.

004: Same here, it's like, to me... I want to say something [INAUDIBLE 35:01] I'm really for it. But, I want to say [INAUDIBLE 35:05] with [INAUDIBLE35:06] restrictions, parents or families do not have any other choice, when I'm vaccinated. But some... Like, let's say my kid is not vaccinated. If I want to go outside of Canada, can I really? So, when they come to this point, I say ‘’OK, I don't have any other choice. I have to’’. So, it's not an option. Yes, for [INAUDIBLE35:31] for my kid, at this age, yes, it's OK. But above 5, I would say ‘’I don't know if it a choice?’’. It's not the matter of choice, you know.

**Yes, for COVID vaccination, we're not in the same situation then for other children vaccine, but they're still recommended or mandatory in Ontario, not in Quebec, but you're right that if you want to travel or if you want to go to a restaurant, then you need to be vaccinated.**

004: It’s not a matter of choice. This is my point. I wanted to say. Also, I'm really for it(36:10), and I know the way out... the benefits outweigh the risks. Why not trying that..? It shows everybody, but at the end, it's not a choice.

**[003] do you want to add something?**

003: No, I'm good.

**Do you feel that it's better when the story characterized... put forward parents, than a healthcare provider? Do you think it's more influential?**

003: I think it was easier to relate to parents, some of what they say... like trying to make the best choices for their children. Trying to decipher what's right and what's wrong. There's a lot of changes over the years very quickly, and where do you get your information. And grandparents cannot give you as accurate information, because they've lived is no longer accurate and stuff like that. So, what it was the question, already?

**It was the difference between having a main character as a healthcare provider versus a parents, and whether one is better than the other?**

003: Yeah. the parents definitely... You can related a bit more to it. Yeah, I answered to the question.

**And is it something that you faced for this negative information about vaccination in your own social media, is that a problem for you in your personal life?**

003: Personally, not as much. I've laid off social media because, indeed, like lots of... [INAUDIBLE38:09] fake stuff flying around now, [INAUDIBLE38:11]. And with my wife being a nurse, I did some schooling around that too, so we were like ‘’yeah, this is misinformation nonstop’’. Even though you know it's not right, when you see it so often, it gets in your head, and starts to influence you. And then, you have to kind of mentally remind yourself ‘’yeah, no, that's not accurate’’. It's weird tough to remind yourself something you really believe in, because you've been exposed so much. So, I laid off a bit social media for that.

**Others, any other opinion?**

001: I think the parents they do... provide a different perspective. They do show that's what they lived through, and they shared their own experiences. For sure, we can related to them more as parents. However, I think they also do need to introduce a doctor or a nurse in that video, as maybe towards to the end, in order to draw a conclusion or a shout out to action ‘’ get your kids vaccinated’’ or something, in the very end.

**For vaccine or other health issue, do you watch a lot of video online? Is that something you do frequently, looking at videos?**

004: Not honestly. I just happen to have one... I don't know if you've seen it, but it's like... Honestly, I don't trust social media, because you find a lot of... always for every subject, no matter what it is, you find a lot supporters and those who are against that. And you cannot really trust what the others thin. You can respect, but it's not the point for making decision, at least for me. For example, do you remember when the... back one year ago, when the vaccine was introduced to the public around... I would say not even one year ago, I would say a couple of months ago. I saw the video that people trying to put fork and a spoon on their body and said that it had magnetic... Something like that...

**I have not seeing this one.**

004: I had seen that and I was really impressed. I tried by my own, and it didn't do the same, honestly. But it was the first time, I want to say I was impressed that much that I trying to do it on my own. And it never happened. So, I was confused. It was only the first time that social media affected my life. Because a lot of unclarity around the [INAUDIBLE 41:41) and it's still... Because every government, every authority, every public elsewhere have different sides of the world. They are saying something different. The main message is saying, but at that time, they said differently, like this one is approved, this one is not approved. This vaccine is approved by WHO. This vaccine is not. But, it's like misinformation. When you have such this misinformation going around, then social media could play a key role in people's mind, somehow.

002: I would like say social media is definitely bad for misinformation as [004] was saying. And that is not just social media, even mainstream media, they play up... every all the negativity is played up, it's amplified. It's repeated 50 times in a newscast. So, I'm not fronting... I've kind of stepped away a bit from that as well. Again, just trusting your sources. Reading an article, looking where this source was given you a big part as well. Clic paid42:57) is everywhere now. I think that's something that attend to look for a bit more like... I definitely want to be informed. I definitely read over something again and review the information, before I make an informed decision.

004: If I can add something to that, if you don't mind. I know I have spoken a lot. But just something coming from a mainstream. I never forgot, two months ago, when [INAUDIBLE43:29] came and said that almost... I don't want to come up with the statistic, but half of the healthcare people... something like that... didn't get vaccinated. So, I just want to say the impact of the media. So, I was just asking myself ‘’ if it's that's helpful, if it's that’s useful, why those who are in the front line, and they are talking about that, not get vaccinated?’’. It's always the question for us. So, it makes things really doubtful. It makes me really doubtful about that. It's good you are publicizing your are advertising this, but why not yourself? And you are in the front lines. Even if we know that it's good for our body. But it's kind of rising the question now, why a lot of nurses at least didn't get vaccinated?

**Yes, that's doesn't send the right messages, but the proportion is quite small, it's less than 10% of nurses you are not been vaccinated. It's not one on two. But, the fact that some healthcare provider does refuse to be vaccinated, it's quite worrying, of course.**

004: Honestly, again, I don't want to come with a static, because I don't know, but it was still a lot of them, 1000 or something like that. I don't know the number. I don't want to say about the numbers, but it was that much peak when they media ask them ‘’OK why don't you stop them from working?’’. It said that ‘’because we are missing 8000 and we're still need them’’. You That's why we take it... and timeline, again. It was in November, December, that’s why. But the main message... I know it's for kids this session, but I just want to say that me as a parent... Yes, I did it for myself, but I've got a question myself one more time. If it's something that is really needed and it's being advertised or something by healthcare system, why those who are involved, are not getting that?

**I hear you. So, you recall more of the fact that some did not than there exact statistic and that's what he strike you and what you remember that's... I think that what we're saying within influence of media, both mainstream and social media, and what people think and do about vaccine.**

004: Right

001: I think that... we are agreeing that the social media they do not always provide the accurate information. And personally, I see social media as a form of entertainment where they don't always provide correct, accurate sources of information. I would go to rather read written articles, in order to get more accurate information that sense. Because it's more serious.

**So, you're not using social media to search for information.**

001: Exactly. Exactly.

**Get you. Anything else on that video before we move to the third story?**

[No answer]

**OK. So, the next one Marie-Eve if you could... So, the last story is about a mother that explain what happened to her kids after contracting a vaccine preventable disease. So, it's a touching story of real parents that talks about why vaccine are important. And her child got really sick from a vaccine preventable disease. So, Marie-Eve if you could put the video on?**

***I think I have a problem with my sound, so I'm just gonna unshare and reshare the video, just wait a minute. I'm going to share it on YouTube because the PowerPoint is not working again.***

***Viewing video # 3 [video mother and child not vaccinated at 48:55 until 53:24]***

**So, first impressions for that video?**

002: That's definitely the video that probably hits the most, closest to home, especially being a parent. I think that's a situation that nobody ever envisions being put in, as sad as it was to see the child in that state, I think that really could probably wake some people up in regards to the importance of all these types of childhood vaccines that are important. To prevent the spread of of illnesses.

003: Similarly, here, it really hit hard in watching this... [INAUDIBLE 54:17] pathetic. And we really don't want that to happen to our child either. Yeah, really helpful (54:23).

**Do you think it's too emotional? Is that playing too much on fear?**

003: Well, when you look at anti vaccine campaigns, they're playing on fear as well. So, we're getting on the same level. It's fear based on a real impact that you can actually happen. They try to minimize it a bit, [INAUDIBLE54:56]... saying it's rare, but it can happen. I wonder if they minimize as much like other videos. I feel like there's a bit of minimization. It happens, but only rarely, it happened, but I was a little late. It almost sounded like she felt guilty, that should not went sooner. I mean by the time he got it, he got it. It's pretty bad, already. Maybe more focusing on [INAUDIBLE 55:37] it happened. I mean it's fair game. Fear works to some... It’s not like you're blowing something out of proportion either, it's a natural inch (55:58) fear. It's something that can actually happen. Is reasonable when you're not vaccinating your child. So, the risk, just portraying it, highlighting it. I think it's fair game.

001: I think the exploits on fear, we see like the striking image of the child who had received more than 50 operations like she said. We can imagine how tough she was to leave all that through. But at the same time, when we back off, we also think about the probability of our own child getting the same disease without vaccine. So, we'll wait, I think. We can wait the benefits on that. I think that's a very strong video that delivered, the mother and the child at the same time on the couch. But it makes me feel sad. Yeah. And we don't want that to happen to any child, that's for sure, but at the same time, we have to... I think we have to... Personally, I agreed to what the mother said, we cannot make some decisions for the child.

**So, often public health hesitates to portray those type of the real bad things that could happen, if you are not vaccinating, but you're right [003] by saying that the anti-vaccine is often put those fear-based tactics forward. So, that's kind of the opposite. But we were a bit unsure about the level of emotion of seeing that and the kind of backfire that could... Some parent could feel by seeing such video.**

001: I think that was a little bit too much.

003: I remember, I was travelling towards Quebec City and I heard on the radio that one adds and it really stuck with me. I think it was from public health, recently. It's about a child who's being abused and they're playing outside the game with the rope. And the first time she's singing really loud and really cheerful and slowly, she's just... her personality becoming more and more depressed and face (59:03) a bit. And they talk about recognizing the sign of abuse and stuff like that. I never forgot. It was so striking. Once they say what it is, it really stuck and it worked really well. So, in that sense, do the same in vaccine, so extent. Get those.

002: Yeah, I think to [003]’s point, I think we need like... As parents, as people, sometimes you need that tough love approach and I'm sure there's a lot of people that they mean to get their child vaccinated for these vaccines and they put it off ‘’I'll get to it. I'll get to it, I'm busy’’. Everyone is busier than they've ever been. So, I think by seeing that and a bit more often, I think it's something that could raise your alarm for a few people, give a motivational kick in the rear to some people to go and get things done. I think, seeing that in a present platform where... To see it on Facebook, you'll just keep scrolling. But if you see it somewhere where... I'm saying YouTube where the add can't skip or somewhere where you can't... where it's really in your face, that it could probably be a good platform to pass those on. But I think, as parents we definitely all need that motivational move from sometime.

**Thanks, do you think there's things missing in that story?**

002: No, I think, honestly, on my part, I think it was really well covered.

004: I also. I believe, our decision could affect the whole life of someone, that has no power. It's like a simple decision can... I don't want to say deteriorate the whole life, but... Honestly, it was really touching me. There was really sensation. It shows the importance of our decision. Not only, getting vaccinated or not. Because the child at this age they cannot make a decision, it's with us. We make a decision and... I don't want to use the word ‘’decision’’, but at least...

001: I think that's a very complete story from the beginning, when the child had 4 months and half. And what happened during the night when he was... the mother discovered him, she called 911 and they got into the hospital. She described in very detailed manner; what the doctors did on him, and while she was standing separated from her baby for more than six hours. And after, we see how the baby growing up. And we will think how he would grow as an adult later on, probably with more health problems later on. I think that leave us to think about the consequences. Not only as a teenager later on, and also as an adult or when he gets old. I mean, health problems that cannot be cured, once at that stage, it's too late, to take any other actions.

**So, coming back to the 3 stories, which one did you prefer?**

001: I prefer the second story. Involved two happy parents... rather happy parents with two children. Very good balance in that representation of parents and children. Versus the first video only one doctor present in the video. And the last, third video, with one mother and one child. Although the second video was the longest, I think he provided a complete story of how...at the first place the parents did not get their proper research done. And did not inconsequence vaccinate their first child. Then afterwards, they got more resources. They researched further and then, they change their mind. I prefer the second story. It's not as sad as the third one and I can relate more to the parents.

**Thanks. I'll do a round table, so which video did you like the least?**

001: I think the first video. As I said, the doctor, although he's a very recognized doctor in the field, but he's still in the park. For the general public, for normal people like us, we don't know who he is. And, what level of authority he has? And we are less connected to... I mean, in the beginning he's interesting, with his point of view, but when he speaks and speaks, then he loses his audience easily like me (laugh).

002: I would say #2 is the most informative. #3 was the most compelling for a call to action. I would, speaking for myself, if I see all three in a situation where my child wasn't vaccinated, that would definitely push me to get out the door. #1 is more... #2 is more, telling a story, kind of their experience as #3, but I said #3 was more compelling to make me want to do something. #1, I just felt it was just more someone who just kind of stating... stating facts and reading, reading a script, essentially not really relatable. So, I would say 2-3-1, for sure.

**Thanks, [003]**

003: I'm really staying between #2 and#3 for the past. I think I wouldn't mind seeing #2 like couple times a week. But #3, I'd see it once a week and that'll be plenty. If I see it too often, I feel overwhelmed and it would lose its impact. I put #2 and #3 really on an equal [INAUDIBLE1:7:24] but not at the same frequency. #2, I could manage a couple times a week. There were a couple problems with #1. #1 was really release (1:07:45)... definitely away from everything. Even when you're thinking, you're wanting people to vaccinate themselves, the people who already did that already believe in the authorities, So, it’s just... I don't think it targets the people were looking for anyway. But #2 and #3 on different level frequency.

004: When I consider [INAUDIBLE1:08:18]... forcing me to get my child vaccinated, I would definitely go with the #3. But for the aspect of... like if I have chance to watch it couple of times, I would definitely go with the number #3, it's really touching. So, I would go with #2. But #2 is really blurry, as I mentioned, it's not pointing the information. So, it depends how we look at it. I'm really between #2 and #3. Because every one of them has its own positive and negative side. But definitely #1 is the least interesting one. That I would have... really, I skip it, if I had the choice. I don't know what's the purpose behind that. [INAUDIBLE1:09:22]. No information, no stimulation. Nothing, behind that.

**Good. And if we want to reach parents like you, what do you think are the best media? Is it the video? Is it a short text with images? Which platform should we target? What length of time are you able to have your attention to something?**

004: To me, short videos are the most effective ones. Giving the main purpose of what we are looking for. Maybe 5 years ago or 10 years ago, it was long videos. But that's why I believe now TikTok videos are really... the public are vibrant or [INAUDIBLE1:10:32]. Because of the effectiveness, the time and the message they are sometimes transferring. So, I would go with short video, but to the point. Make it really, really to the point. Max one minute, or even less.

**One-minute max.**

004: Yes.

**Good. Other views.**

001: I would say the same thing: one-minute max. Personally, I use... I don't use TikTok. I don't mind longer videos, but I prefer shorter ones. I use YouTube and Facebook, mostly. I would like to see the ads on those platforms and maybe have two versions. 1 version with a very short video within maybe 20 or 30 seconds. And then, you have a longer one with a lot of more information there. That video could lasts 2 or 3 minutes.

003: For me, Facebook and YouTube as well, definitely would be the main targets. But what I'd say when you start the video, you got about 5 second to really catch my attention. Otherwise, even if your video is10 seconds, I'm not going to watch it all. The first 5 seconds are crucial for me. If you're not saying something or catching me within that time... Sometime had ads that caught me within those five seconds, and they're two-minute long, and I only notice it's an add a minute and a half later. But usually between 30 seconds and 1 minute, I'll probably watch the ad, if it's good enough. If it's making strong message or it's something interesting. Facebook, definitely for my wife.

002: Yeah, I would agree with the group. I think, TikTok is good. You're seeing a lot more exposure out there by mainstream companies. So, it's definitely a good network to get a message passed along. The other good convenience you're interested, you want to see more, it's all kind of in one spot. So, you can just look in. So, I would say for short videos. And a they can tell a story through there by... in segments and all. Facebook, for ads, I think they kind of get lost in translation and people just keep swiping. They'll just keep going until they see something they want to see. So, I think that could be lost in that network a bit. And then, You Tube, ad advertise ads on YouTube, I think that's one that... Believe it or not, I'm actually realizing a lot more is on Spotify. Spotify if you're not a premium member, whatever if you're stuck listening to those. Honestly, Spotify, I would say is really one that gets my attention a lot more for ads. Because you're stuck listening to him. So, you're there... I'm sure I'll be cursing and hearing and laugh after 20^th^ time, or whatever. But for a quick hook (1:14:28). One caution still not over indulging people, over advertiser, but that's a really good network for people to get a message through on that platform.

003: I definitely agree with [002] on that with Spotify. Even my wife made a comment out of nowhere.

**So, we should aim to target places where people are forced to listen to it. And at be really catching the first 5 second, before people are able to skip ad.**

002: Yeah, like Spotify you could do 30 seconds, one or more. ‘’How to protect your children, come here, [INAUDIBLE 1:15:13] maybe you get them on board.

**Great, I'm looking at my question. We were wondering at the beginning, another story that we didn't show could be effective. One, it’s a parent... a bit like the second one appearance who did not vaccinated, explain why in details, they finally decide for vaccination, like a typical conversion story of a parent with anti-vaccine that become pro-vaccine, and finally accept vaccination. Is that something that you think it would be effective?**

004: I believe yes is the most effective one for me, like what’s change? That's I was like I was asking for those who are influenced. What make them to change their mind? That's really important. That’s the point, I believe, that's the main point of the these all videos.

002: Maybe combining video #2 and #3 into one. ‘’Here's my story. I was an anti-vaxxer. I regret it because X things happened to my kids’’. I think you can marry those two together. And I think, that could be a compelling case, for sure.

**Great. Anything else you want to share before we wrap up?**

004: Not from my side, thank you.

**So, thanks so much for your time tonight. It's really appreciated. If we develop other videos, are you willing for us to share them with you by via email, so we could provide some short feedback on what you think about those videos and whether you like them or not?**

002: Yeah, for sure.

**If you don't want, you just don't have to reply back, and we won't arrest you with that.**

002: Just be like Spotify and not every day. (Laugh)

**And again, thank you, it was really a pleasure to meet you all, tonight. Wishing you all the best for the coming.**

002: Alright thank you. Bye bye. Have a good night.

**Bye bye.**
