# Supplementary material for: Exploring parents’ views of the use of narratives to promote childhood vaccination online
Source: PLoS One. 2023 Jul 19;18(7):e0284107. doi: 10.1371/journal.pone.0284107 (PMC10355395; doi:10.1371/journal.pone.0284107)
Supplement: Attachment — Submitted filename: Letter of response_Oct7.docx [file pone.0284107.s004.docx]

Emily Chenette

Editor in chief

PLOS ONE

**Revision of the manuscript: [PONE-D-22-21909] - [EMID: fedcde29c44b44db]**

Dear editor,

We thank the reviewer for their comments and feedback. Please find below our responses and the corresponding changes. All changes in the manuscript were done using the track change mode and a clean version is also submitted. We have made some changes in the manuscript according to your comments and the revisors comments. We also made minor clarifications in the manuscript to ensure readers comprehension. See below our answers to the comments.

ACADEMIC EDITOR:

1. The research participants and/or authors who were responsible for content /video creation should be specified.

*Answer: Videos used for this project were not created for our specific project but were identified through the first phase of our project. The videos were all publicly available on YouTube and Facebook. For the paediatrician story: Arnaud Gagneur from Sherbrooke University for the French version ( https://www.youtube.com/watch?v=B_kpGPZeShI) and The Children’s Hospital of Philadelphia for the English version (https://www.youtube.com/watch?v=xNbVjCdvrdA ). For the informed decision-making videos featuring parents: Kids Boost Immunity (https://www.facebook.com/iboostimmunity/posts/1432957773404880/)*. For the mother’s experience with vaccine preventable disease: Immunize Canada (https://www.youtube.com/watch?v=mWPZGYAdcnc ).

*We added information in the methods about the videos (The links to the videos used during focus group discussion are available in supporting information 1)*

2. Authors should also indicate if translations were made by professional translators. Please address the questions, comments and concerns from each reviewer.

*Answer: We added this information on line 139:* *For the paediatrician story video, we found a French video and an English video with a similar story. For the informed decision-making video featuring parents, the original video was in English and we added French subtitles with the help of a bilingual trainee. English subtitles were already available for the video of the mother’s experience with vaccine preventable disease. For data collection, focus groups were conducted in both French and English and transcribed in original language. The analysis was conducted with verbatim in their original language. All quotes in French were translated by the authors for the manuscript.*

3. We note that you have provided funding information that is not currently declared in your Funding Statement. However, funding information should not appear in the Acknowledgments section or other areas of your manuscript. We will only publish funding information present in the Funding Statement section of the online submission form. Please remove any funding-related text from the manuscript and let us know how you would like to update your Funding Statement. Currently, your Funding Statement reads as follows:
   "This research was funded by the Canadian Institutes of Health Research Catalyst Grant. ED received the grant. https://cihr-irsc.gc.ca/f/193.html The funders had no role in study design, data collection and analysis, decision to publish, or preparation of the manuscript." Please include your amended statements within your cover letter; we will change the online submission form on your behalf.

*Answer: We removed the funding statement in the acknowledgments section and the funding section. This should go in the funding section: “**This research was funded by the Canadian Institutes of Health Research Catalyst Grant. ED received the grant. https://cihr-irsc.gc.ca/f/193.html The funders had no role in study design, data collection and analysis, decision to publish, or preparation of the manuscript."*

a) If there are ethical or legal restrictions on sharing a de-identified data set, please explain them in detail (e.g., data contain potentially sensitive information, data are owned by a third-party organization, etc.) and who has imposed them (e.g., an ethics committee). Please also provide contact information for a data access committee, ethics committee, or other institutional body to which data requests may be sent.

b) If there are no restrictions, please upload the minimal anonymized data set necessary to replicate your study findings as either Supporting Information files or to a stable, public repository and provide us with the relevant URLs, DOIs, or accession numbers. For a list of acceptable repositories, please see http://journals.plos.org/plosone/s/data-availability#loc-recommended-repositories.

We will update your Data Availability statement on your behalf to reflect the information you provide.

*Answer: After consultation of our institution research ethics board, we submitted a minimal anonymized data set as a Supporting information file 3.*

4. PLOS requires an ORCID iD for the corresponding author in Editorial Manager on papers submitted after December 6th, 2016. Please ensure that you have an ORCID iD and that it is validated in Editorial Manager. To do this, go to ‘Update my Information’ (in the upper left-hand corner of the main menu), and click on the Fetch/Validate link next to the ORCID field. This will take you to the ORCID site and allow you to create a new iD or authenticate a pre-existing iD in Editorial Manager. Please see the following video for instructions on linking an ORCID iD to your Editorial Manager account: <https://www.youtube.com/watch?v=_xcclfuvtxQ>.

*Answer: we added the ORCID ID.*

Your ethics statement should only appear in the Methods section of your manuscript. If your ethics statement is written in any section besides the Methods, please move it to the Methods section and delete it from any other section. Please ensure that your ethics statement is included in your manuscript, as the ethics statement entered into the online submission form will not be published alongside your manuscript.

*Answer: We have put the ethics section in the methods.*

5. Please include captions for your Supporting Information files at the end of your manuscript, and update any in-text citations to match accordingly. Please see our Supporting Information guidelines for more information: <http://journals.plos.org/plosone/s/supporting-information>.

*Answer: We added the information on line 173:” (see supporting information 2 for interview guide).”*

6. Please review your reference list to ensure that it is complete and correct. If you have cited papers that have been retracted, please include the rationale for doing so in the manuscript text, or remove these references and replace them with relevant current references. Any changes to the reference list should be mentioned in the rebuttal letter that accompanies your revised manuscript. If you need to cite a retracted article, indicate the article’s retracted status in the References list and also include a citation and full reference for the retraction notice.

*Answer: We reviewed the reference list.*

7. Reviewer#1 The discussion of COVID 19 vaccines is a bit of a red herring, especially as most studies discussed are not in the context of childhood vaccination. Suggest focusing mainly on childhood vaccines as it's important to do this work regardless of COVID! The authors situate this study within a broader one.

*Answer: We agree with the reviewer’s comment and our project started prior to the pandemic. Sentences related to COVID-19 pandemic and vaccines were removed to avoid confusion.*

8. As such, I think they could emphasise that they chose existing narratives for expediency. The fact that they analysed these narratives to draw out commonalities and main themes before choosing exemplars is good. However, I think they also needed to consider where these videos were stored and saved and who made them. Might this matter to the viewers? Was this discussed in the FGs? If not, this is a limitation of the study.

*Answer: As noted in response to the Editor’s comment Videos used for this project were not created for our specific project but were identified through the first phase of our project. The videos were all publicly available on YouTube and Facebook. For the paediatrician story: Arnaud Gagneur from Sherbrooke University for the French version (*[*https://www.youtube.com/watch?v=B_kpGPZeShI*](https://www.youtube.com/watch?v=B_kpGPZeShI) *) and The Children’s Hospital of Philadelphia for the English version (*[*https://www.youtube.com/watch?v=xNbVjCdvrdA*](https://www.youtube.com/watch?v=xNbVjCdvrdA) *). For the informed decision-making videos featuring parents: Kids Boost Immunity (*[*https://www.facebook.com/iboostimmunity/posts/1432957773404880/*](https://www.facebook.com/iboostimmunity/posts/1432957773404880/) *). For the mother’s experience with vaccine preventable disease: Immunize Canada (*[*https://www.youtube.com/watch?v=mWPZGYAdcnc*](https://www.youtube.com/watch?v=mWPZGYAdcnc) *).*

*We added information in the methods about the videos (The links to the videos used during focus group discussion are available in supporting information 1)*

*We also added on line 136: “Subsequently, for expediency an online search was conducted to identify narratives (in video format) that counter the most recurrent negative themes that were identified, as described in Table 1.”*

9. The other issue was about language. It wasn't evident that all videos were in both languages, even though the authors said they were. Please be clear and transparent about this. It seems that for videos to exist in both languages they'd need to be Canadian but this also wasn't self evident. Also, with some data collected in English and some in French, when was it translated, how, and by whom? At what point in the analysis?

*Answer: As noted in response to the Editor’s comment: We added this information on line 138: For the paediatrician story video, we found a French video and an English video with a similar story. For the informed decision-making video featuring parents, the video was in English and we added French subtitles with the help of a bilingual undergraduate student English subtitles were already available for the video of the mother’s experience with vaccine preventable disease. For data collection, focus groups were conducted in both French and English and transcribed in original language. The analysis was conducted with verbatim in their original language. All quotes in French were translated by the authors for the manuscript.*

10. In the results, the authors could lean more into whether the particularly Vax hesitant people said different things to the other participants.

*Answer: Only two participants overtly expressed their hesitation toward vaccination during the focus groups and their opinions on the videos were different (i.e., one participant really appreciated the mother’s video while the other disliked it). We added a sentence in the limitations.*

11. Also the use of 'blouse' is weird - blouses are specifically a type of women's shirt. Do you mean shirt?

*Answer: We changed the word “blouse” for “smock” as we were referring to doctors and nurses usual wearings.*

12. When people, at line 223, think "this is not an important issue", what do you mean by "this"?

*Answer: We changed “this” for “having difficulties to find information online”*

13. I think a really interesting finding from this study is that government probably needs to pay for the team's eventual narratives to be disseminated. People won't go looking for them. Suggest leaning into this more.

*Answer: Line 357 we added: “**As some participants suggested, it could be beneficial for online narratives in a video format to feature on governmental pages about vaccine information to make a decision. This could facilitate dissemination of narratives-based vaccine promotion strategies. The feasibility of this avenue should be explored as past analysis have highlighted that official websites are generally not using such approaches.”*

Reviewer #2:

14. Table 1: Can the authors please clarify what the ‘n’s represent in this table? For example, for ‘negative narratives (n=26)’, does this indicate that 26 different negative stories were identified even if some of these stories appeared in different forms across different platforms (meaning maybe >26 unique videos, posts, articles were found with some duplicate content), OR does this indicate that 26 unique online content pieces were identified and a Facebook post about a specific myth would be counted separately from a Tik Tok video with a creator repeating that same myth. I recognize this is a nuanced question. I think understanding whether the study team viewed a total of 37 pieces of online content vs >37 pieces of content that all referenced back to about 37 narratives would help the reader contextualize the extent of the search used to identify narratives.

*Answer: The n referred to the number of videos that were analyzed for each category (negative, neutral, positive). We agree with reviewer’s comment that this information does not add much to the manuscript and have removed the n of the table.*

Data collection sub-section: I think the last sentence of this paragraph should refer to the focus group guide (not interview grid) being available in appendix 1. The interview grid does appear as Figure 1, just as the authors have stated in the first sentence of this paragraph. Suggest rewording, or if I have misinterpreted then I’d suggest the authors provide more clear definition of what is meant by ‘interview grid’ vs ‘focus group guide’

*Answer: We changed interview grid for focus group guide everywhere in the manuscript. We added on line 173: (Focus group guide is available in supporting information 2)*

15. Minor comment: In the references, the dates for publication are in French whereas the rest of the text is in English. Suggest changing reference language format for consistency.

*Answer: We have reviewed all the references to make sure it is up to date and in English.*

16. Results: Can the authors provide any context about having 3 focus groups with French-speaking parents and 1 with English-speaking parents? Is this generally representative of the population in Quebec? Or is it aligned with know demographics associated with vaccine hesitancy in Quebec?

*Answer: The majority of Quebecers are having French as their first language and 13.7% English. We added a sentence in the Methods line 182: “**The repartition of English and French focus group discussion reflects Quebec’s language demographic as the majority use French as their main language (86.3%). Although the majority of Quebecers speak French, we conducted focus group discussion with anglophones to explore potential cultural – linguistic differences.”*

17. Table 2: Please provide a legend or footnote indicating the meaning of the asterisk included after the heading ‘Level of hesitancy’.

*Answer: We removed the asterisk and clarified the hesitancy scale in the main text line 209: “Level of hesitancy was determined according* *to self-evaluation using a 10-point scale. “*

18. ‘Parents’ opinions about childhood vaccination in general’ subsection: This paragraph notes that participants had very positive attitudes toward vaccines, but Table 2 notes six parents at having ‘high’ vaccine hesitancy and others with moderate hesitancy. Can authors please provide some comment to reconcile these two findings? Is information in table derived from survey scores and information in the text based on qualitative responses?

*Answer: Line 218 we changed “all” for “many”.*

19. Discussion
    Third paragraph: The sentences that begin ‘This finding highlight that…’ and ‘Finally, the literature is still unclear in the literature’ are both difficult to read due to problems with subject/verb agreement or possibly typographic errors. I’d ask the authors or one of the editors to review these two sentences closely to revise and improve clarity/readability.

*Answer: Line 314: We changed “this finding highlight that” for “Optimizing the benefits of using videos for vaccine promotion, it is of critical importance to ensure to have a captive audience when designing the intervention.” Line 303: we removed: “the literature is still unclear” to avoid repetitions.*

Reviewer #3:

20. As stated by the authors (line 197), most participants held positive views of vaccines, and (line 201) intended to or already had vaccinated their children. This is a limitation that should be mentioned more clearly in the discussion and/or limitations section.

*Answer: We added on line 338: “The fact that most participants were positive about vaccination and that majority of their children were already immunized is a major limitation to understand views of hesitant parents about the videos.”*

21. In addition to mentioning the inability to recruit vaccine-hesitant parents to the study, authors should (in the discussion) provide a few sentences about how previous researchers went about recruiting vaccine-hesitant parents ( = the target group of the proposed online narratives), and what approach the authors suggest in future studies to reach/recruit vaccine-hesitant parents.

*Answer: We added this sentence in the limitations, line 342: Recruitment via social medias using a question to filter non-hesitant parents has been shown to be an effective way of recruiting vaccine hesitant parents(43).*

22. Debunking false vaccine information by health care providers and public health authorities has repeatedly been shown to be ineffective in changing vaccine-hesitant parents minds. In light of this, authors should cite these negative studies and discuss how such online narratives might be adapted to better reach vaccine-hesitant parents.

*Answer: We added precisions, line 354:* *Although recent studies have shown positive impact of debunking myths on vaccination acceptance, these approaches can backfire for the highly hesitant (43,44). By promoting messages on the importance of vaccination to prevent disease instead of focusing on specific myths, the use of narratives is a promising approach.*
